# Evolutionary principles guiding amphibian conservation

**DOI:** 10.1111/eva.12940

**Published:** 2020-03-13

**Authors:** Maciej Pabijan, Gemma Palomar, Bernardo Antunes, Weronika Antoł, Piotr Zieliński, Wiesław Babik

**Affiliations:** ^1^ Institute of Zoology and Biomedical Research Faculty of Biology Jagiellonian University Kraków Poland; ^2^ Institute of Environmental Sciences Faculty of Biology Jagiellonian University Kraków Poland

**Keywords:** amphibians, conservation biology, conservation genetics, habitat degradation, host parasite interactions, natural selection and contemporary evolution

## Abstract

The Anthropocene has witnessed catastrophic amphibian declines across the globe. A multitude of new, primarily human‐induced drivers of decline may lead to extinction, but can also push species onto novel evolutionary trajectories. If these are recognized by amphibian biologists, they can be engaged in conservation actions. Here, we summarize how principles stemming from evolutionary concepts have been applied for conservation purposes, and address emerging ideas at the vanguard of amphibian conservation science. In particular, we examine the consequences of increased drift and inbreeding in small populations and their implications for practical conservation. We then review studies of connectivity between populations at the landscape level, which have emphasized the limiting influence of anthropogenic structures and degraded habitat on genetic cohesion. The rapid pace of environmental changes leads to the central question of whether amphibian populations can cope either by adapting to new conditions or by shifting their ranges. We gloomily conclude that extinction seems far more likely than adaptation or range shifts for most species. That said, conservation strategies employing evolutionary principles, such as selective breeding, introduction of adaptive variants through translocations, ecosystem interventions aimed at decreasing phenotype–environment mismatch, or genetic engineering, may effectively counter amphibian decline in some areas or for some species. The spread of invasive species and infectious diseases has often had disastrous consequences, but has also provided some premier examples of rapid evolution with conservation implications. Much can be done in terms of setting aside valuable amphibian habitat that should encompass both natural and agricultural areas, as well as designing protected areas to maximize the phylogenetic and functional diversity of the amphibian community. We conclude that an explicit consideration and application of evolutionary principles, although certainly not a silver bullet, should increase effectiveness of amphibian conservation in both the short and long term.

## 1 INTRODUCTION

The Anthropocene has witnessed the demise of many amphibian populations across the globe (Cushman, 2006). Approximately 40% of extant amphibian species are threatened with extinction (IUCN Red List Data version 2019‐1) and 34‐170 are likely already extinct, at least in the wild. In addition, species with insufficient data for assessment (~21% of assessed species) are predicted to be more threatened with extinction than previously recognized (González‐del‐Pliego et al., 2019).

Extant amphibians live in environments exposed to a multitude of primarily human‐induced drivers of decline. These drivers have been exhaustively reviewed elsewhere (e.g., Collins & Crump, 2009) and are briefly summarized in Box 1. Apart from causing demographic crashes and extinction, these stressors have a more nuanced effect on amphibian populations by inducing phenotypic and evolutionary change. For instance, adaptation to changing conditions might mitigate some of the detrimental effects of environmental degradation (Flynn, Love, Coleman, & Lance, 2019). Pathogens may remodel amphibian communities by altering biotic interactions among species with different levels of susceptibility (Bosch & Rincón, 2008). Climate change has allowed some species to colonize previously unavailable habitat or expand demographically in some areas (Bosch, Fernández‐Beaskoetxea, Garner, & Carrascal, 2018). Human encroachment has created new types of habitat (artificial water bodies, canals, rice paddies) that can be colonized by a few species that can adapt to and exploit altered environmental conditions (e.g., Davies, Hill, McGeoch, & Clusella‐Trullas, 2019). Some of these examples, and others mentioned further in the text, reflect evolution in action in current amphibian populations. The challenge for amphibian conservationists is to manage the evolution of natural populations in a way that will secure their fate into the future.

BOX 1 **The complexity of amphibian declines**

**Figure 1:**
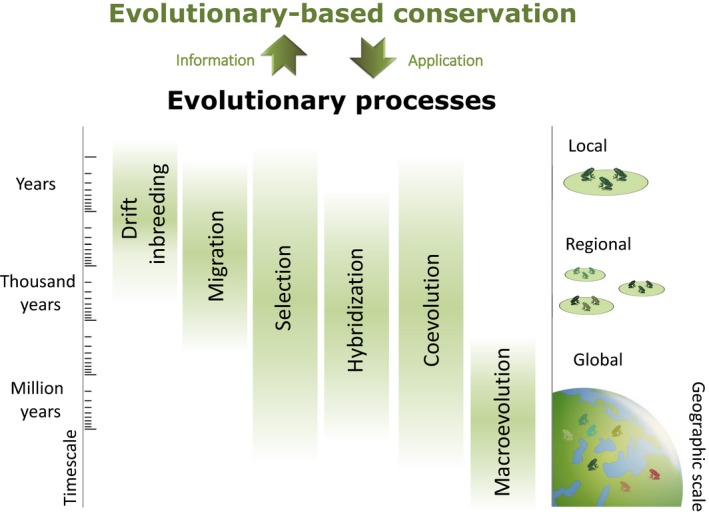
The framework of evolutionary conservation integrates processes operating at various scales

No single remedy for the global amphibian decline exists because it is driven by a multitude of interacting factors (Figure 1, reviewed in Blaustein & Kiesecker, 2002; Blaustein et al., 2018; Collins & Crump, 2009; Cushman, 2006). Degradation or *destruction of habitat* due to changes in land use is probably the foremost threat to amphibians. The spread and outbreak of *emerging infectious diseases*, mostly by chytrid fungi and ranaviruses, but also trematodes *Ribeiroia ondatraeis*, fungi *Saprolegnia ferax*, and protists *Amphibiocystidium ranae*, have triggered widespread declines and commanded the attention of conservation biologists in recent decades. The introduction of *harmful chemicals* (fertilizers, pesticides, and other environmental contaminants) into the world's air, soil, and particularly water supply alters development and physiology of amphibians and increases mortality. Furthermore, pollution can compromise host immune response or its protective microbiome, increasing pathogen virulence (Jiménez & Sommer, 2017). The introduction of *alien species* that predate, compete, hybridize with, or spread infectious diseases in autochthonous amphibian populations (Kraus, 2008) has taken a heavy toll on amphibian communities. A factor that is usually ignored in studies on amphibian declines is the role of population density and community composition. High densities increase not only competition but also the probability of infection. The natural composition of amphibian communities affects predation, competition, hybridization, and spread of emerging diseases, although higher diversity of an assembly can have a dilution effect on pathogens. In addition, the local or regional causes of decline are exasperated by global stressors. Rapid changes in temperature and precipitation patterns due to *global change* are affecting amphibians by shifting ranges and modifying life‐history traits as well as biological and ecological interactions. For instance, climate change can increase temperature up to the optimum of pathogen development triggering a disease outbreak (Bosch, Carrascal, Duran, Walker, & Fisher, 2007). Desiccation caused by climate change can elevate levels of UV‐B radiation in shallow waters increasing the effect of *Saprolegnia* fungus and environmental changes, and contamination can act as a cofactor influencing chytrid outbreaks in tropical America. Amphibians are overharvested for consumption and trade. The *global trade* in amphibians is causing or at least facilitating disease transmission and the establishment of non‐native species. In conclusion, the global and local drivers of amphibian declines seldom act single‐handedly. Instead, many amphibian populations are under the influence of two or more factors at once and the synergistic interactions between them oftentimes prove deadly to susceptible species (e.g., Cohen, Civitello, Venesky, McMahon, & Rohr, 2019). Despite their important role in amphibian decline, these interactions are poorly understood and, since the severity of each of the drivers varies in space and time, their interactions also vary. Hence, the main challenge for amphibian conservation is to find solutions to a local–global phenomenon occurring at multiple scales, identifying the specific drivers and their complex interactions, and dealing with them at the appropriate spatial scale (Grant et al., 2016).

The need to incorporate evolutionary considerations to understand and mitigate amphibian declines was clearly voiced more than a decade ago (Blaustein & Bancroft, 2007). Several reviews, particularly those focusing on the use of molecular markers, discussed evolutionary aspects of amphibian conservation (e.g., Allentoft & O’Brien, 2010; Beebee, 2005; McCartney‐Melstad & Shaffer, 2015; Shaffer et al., 2015). However, an overview structured according to the major evolutionary mechanisms in a conservation context is unavailable and we hope to fill this gap. In this review, we examine how major evolutionary mechanisms relate to current amphibian population status (current threats) and what they mean for practical amphibian conservation. We summarize how evolutionary principles have been applied in amphibian conservation, identify gaps, and provide recommendations as to where the application of evolutionary principles is likely to provide immediate and long‐term progress in practical conservation. Our goal is to discuss how (and if) evolutionary mechanisms can be exploited toward the benefit of declining amphibian populations. Two important themes permeate this review. First, it is becoming widely appreciated that ecological and evolutionary processes are coupled at temporal scales that are relevant for conservation (Hendry, 2016; Stockwell, Hendry, & Kinnison, 2003). One pertinent amphibian example implicates an evolutionary response in the Sierra Nevada yellow‐legged frog (*Rana sierra*) involving reduced susceptibility to the frog‐killing chytrid fungus (Knapp et al., 2016). The remarkable recovery of this species in Yosemite National Park after decades of decline implies that evolutionary changes in imperiled amphibian species may occur at temporal and spatial scales compatible with human‐aided recovery programs. Second, the field of applied evolution has emerged, with the aim of harnessing evolutionary processes to address global challenges, including biodiversity conservation (Carroll et al., 2014), providing a useful framework to conceptualize conservation work in light of evolutionary principles.

**Figure 2:**
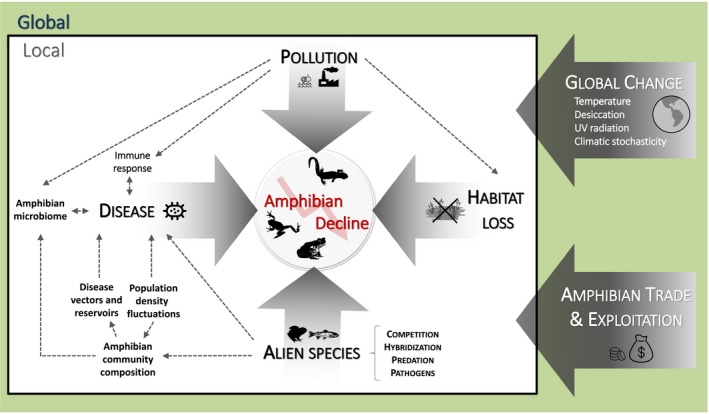
Drivers of amphibian decline and their complex interactions

This review is structured according to evolutionary mechanisms and processes pertinent to amphibian conservation, starting at the scale of generations in local populations and ending with macroevolutionary processes (Figure 2). We emphasize factors that dictate population genetics (e.g., migration, drift, and selection), reflecting the large body of literature on the subject, but also highlight the effects of species interactions (invasives versus locals, pathogens versus hosts, hybridization) and macroevolutionary patterns meaningful for conservation. A notable exception is the lack of a section on mutation—because pressure from de novo mutations per se is unlikely to be of relevance at the temporal scale of a typical conservation horizon. We discuss, however, mechanisms affecting the dynamics of mutations segregating in populations, as these are important for conservation.

## 2 DRIFT AND INBREEDING

Understanding the evolutionary effects of reduction in population size has been a major goal in conservation biology (Frankham, Ballou, & Briscoe, 2010). The main consequences of increased drift in small populations, collectively termed “genetic erosion,” are loss of genetic variation and increased inbreeding. The former leads to the loss of adaptive potential, while the latter exposes the phenotypic effects of deleterious recessive mutations, leading to inbreeding depression. The effective population size (*Ne*, Box 2), which measures the strength of drift, is a key parameter of conservation relevance (Hoffmann, Sgrò, & Kristensen, 2017) that generally negatively correlates with extinction risk (Frankham, 2005).

BOX 2 **Estimation and estimates of *Ne* in amphibians**

Effective population size (*Ne*) is usually lower than census size and depends on sex ratio among breeders, variance in reproductive success between individuals, and, crucially, population size fluctuations across generations. Probably most relevant for conservation is contemporary, current, or recent (several generations) *Ne* of individual populations. These *Ne* estimates in amphibians usually go in tens, and only rarely in hundreds or thousands of individuals (Phillipsen, Funk, Hoffman, Monsen, & Blouin, 2011; Schmeller & Merilä, 2007). The ratio of *Ne*/*N* varies from <0.01 to close to 1, but often is relatively high. Interestingly, elevated *Ne*/*N* ratios have been reported for small amphibian populations (Álvarez, Lourenço, Oro, & Velo‐Antón, 2015; Beebee, 2009). The mechanisms behind genetic compensation of this sort are poorly understood but may involve reduced competition between males (Beebee, 2009) and have also been linked to intraspecific variation in phenotypic traits such as body size (Coles, Reading, & Jehle, 2019). Contemporary *Ne* is usually estimated using molecular markers. Historically, approaches based on temporal changes of allele frequency have been popular, but recently single‐sample methods that explore linkage disequilibrium among unlinked markers or relatedness among individuals in a sample tend to dominate (Wang, Santiago, & Caballero, 2016).

Modest contemporary *Ne* values contrast markedly with estimates of tens or hundreds of thousands of individuals inferred from nucleotide variation using evolutionary models (e.g., Pabijan, Zieliński, Dudek, Stuglik, & Babik, 2017). Species‐wide effective population size is conveniently defined in terms of the expected time to coalescence in a sample of DNA sequences (Charlesworth & Charlesworth, 2010). Such defined metapopulation *Ne* depends not only on population sizes but also on migration between them and can greatly exceed the sum of the census population sizes if migration between populations is low. To make things more complicated, estimates depend critically on the way DNA sequences are sampled. For example, under the assumption of a large number of populations, to estimate species‐wide *Ne*, a single sequence per locus should be sampled from multiple populations. A sum of (long‐term) effective sizes of a number of populations connected by migration can be inferred from DNA sequences sampled from a single population. The distinction between different aspects and timescales of *Ne* is well understood in the population genetic literature, but not sufficiently appreciated in the conservation context. Thus, paradoxically, a highly subdivided species harboring as a whole substantial genetic variation and characterized by large species‐wide *Ne* can actually consist of very small populations on the verge of extinction.

Fragmented populations of widespread European anurans show loss of genetic diversity and reduced fitness (Johansson, Primmer, & Merilä, 2007; Luquet et al., 2011; Rowe & Beebee, 2003). However, heterozygosity–fitness correlations (HFCs) at the individual level have been observed only in a subset of amphibian studies (Allentoft & O’Brien, 2010). This is expected because HFCs, reflecting the effect of inbreeding depression, are only detectable in cases when substantial variance in inbreeding occurs between individuals. Two sources of reduced fitness due to deleterious mutations can be distinguished: (a) fixation of slightly deleterious mutations causing fixation load detectable by interpopulation comparisons and (b) the effects of strongly deleterious recessive mutations that segregate in populations and are exposed by inbreeding, detectable as HFCs at the individual level. In the European tree frog (*Hyla arborea*), larval fitness in small fragmented populations suffered from fixation load but HFCs were undetectable (Luquet et al., 2011). Moreover, neither fixation load nor HFCs were detected for adults in the wild, indicating either differences between traits in sensitivity to the accumulation of deleterious mutations, or the confounding effect of environmental variation (Luquet et al., 2013). The prediction that inbreeding depression should be more severe in the wild was corroborated by finding HFCs in the wood frog (*Lithobates sylvaticus*) in the field but not in the laboratory (Halverson, Skelly, & Caccone, 2006). If inbreeding depression is manifested at the larval stage, many pond‐breeding amphibians characterized by high fecundity and high larval mortality may purge deleterious mutations without an excessive demographic cost of selection. Evidence for strong selection against inbreeding acting at the larval stage was found in a small population of the Italian agile frog (*Rana latastei*) (Ficetola, Garner, Wang, & De Bernardi, 2011). This mechanism may buffer the negative effects of small population size, allowing quick population recovery following habitat restoration. In another study on *R. latastei*, Ficetola, Garner, and De Bernardi (2007) showed that while variation within populations was affected by both habitat fragmentation and distance from the glacial refugium, fitness declined as a function of the former but not the latter. It is thus important to distinguish between old and recent reductions in variation, as their consequences for population viability and conservation may differ.

Threatened amphibians often have low census and effective population sizes, but *Ne*s’ of local populations are naturally small in many widespread species (Box 2). Small populations of threatened amphibians may nonetheless harbor levels of genetic variation comparable to those of larger populations of the same or related species. For example, the ancient and highly endangered Hula painted frog (*Latonia nigriventer*), with contemporary *Ne* of several tens of individuals, still retains substantial microsatellite variation (Perl et al., 2018). Detailed historical records of fire salamander (*Salamandra salamandra*) occurrence in Oviedo, Spain, showed that despite isolation for hundreds of generations, populations in the city center still harbor substantial genetic variation (Lourenço, Álvarez, Wang, & Velo‐Antón, 2017). Even in small populations, the loss of genetic variation can be a lengthy process, so a time lag is expected before the reduction of variation becomes apparent. Nevertheless, examples of endangered species that probably have always been small and isolated, such as the Montseny brook newt (*Calotriton arnoldi*) (Valbuena‐Ureña, Soler‐Membrives, Steinfartz, Orozco‐terWengel, & Carranza, 2017), indicate that even those can exhibit substantial variation.

A potentially highly effective conservation intervention alleviating the adverse genetic effects of reduced *Ne* is genetic rescue, involving the introduction of individuals from a different gene pool to improve fitness in the recipient population (Frankham, 2015). Surprisingly, we could not find any well‐documented reports of genetic rescue in amphibians, despite strong advocacy of the approach (Beauclerc, Johnson, & White, 2010). Beebee (2018) described a possible case of apparent genetic rescue in a peripheral natterjack toad (*Epidalea calamita*) population in the UK due to the inadvertent release of tadpoles from another population.

A recent example of using the principles described in this section for conservation is the case of the critically endangered dusky gopher frog (*Lithobates sevosus*). The implementation of intense conservation efforts, including a headstarting program (raising hatchlings or juveniles in captivity and then releasing them into the wild) in 2002, resulted in an increase in *Ne*, as assessed using microsatellite markers, over a 17‐year period (Hinkson & Richter, 2016), suggesting the effectiveness of management practices.

### 2.1 Implications for conservation

Strong links exist between demography, evolutionary potential, and conservation. In recent years, our understanding of *Ne* in amphibians has improved, with the crucial insight that *Ne* of local populations is often modest (Box 2), although more data from directly developing tropical taxa are needed. Nevertheless, genetic variation may be considerable even in small populations. It appears that the effect of fragmentation on genetic variation is stronger in widespread species with high population connectivity than in naturally highly subdivided species. It is possible that in the latter, populations are largely independent in the medium term, so the effects of increased isolation are only gradually manifested and not yet clearly visible. Therefore, it is important to distinguish between recent fragmentation in high gene flow species and historical fragmentation of species of naturally low connectivity. Conservation actions improving connectivity may be of more immediate effect in the former, but are also important for the latter in the long term. In any case, the success of conservation interventions may be assessed by following temporal trends in *Ne* using available tools (Boxes 2 and 3). Cautious optimism regarding the prospects of small amphibian populations is warranted because of genetic compensation (Box 2) and potential for selection against inbreeding without excessive demographic cost. To increase *Ne* in captive colonies even above census size, breeding schemes such as equalizing the number of progeny between families or subdivision can be used (Wang et al., 2016).

BOX 3 **An expanding molecular toolbox aids amphibian conservation**

Conservation efforts can be severely hampered by incomplete distributional and taxonomic information. The proportion of unknown or undescribed amphibian taxa, particularly in tropical communities, can be staggering (Vieites et al., 2009), and some groups are particularly deficient in relevant conservation data (e.g., caecilians, Gower & Wilkinson, 2005). Molecular assessments of diversity, using rule‐of‐thumb sequence divergence thresholds in mtDNA, can be applied to screen for candidate species to be confirmed by integrative taxonomy approaches. Environmental DNA (eDNA) has emerged as a complementary or even alternative tool for monitoring amphibian species composition and has been particularly successful for secretive and rare species (e.g., hellbenders, Wineland et al., 2019). With improvements in sampling design (Goldberg, Strickler, & Fremier, 2018) and proof‐of‐concept studies for tropical batrachofauna (Bálint et al., 2018), eDNA metabarcoding has the potential to advance biodiversity assessment as well as long‐term monitoring over large spatial scales. Community metabarcoding is also used to study diversity of amphibian microbiomes and has applications in disease mitigation and captive breeding for reintroduction purposes (Jiménez & Sommer, 2017).

Although full genomes are still a rare commodity in amphibian research, genome‐scale molecular data are now accessible through the application of reduced representation techniques such as transcriptome sequencing, restriction‐site‐associated DNA sequencing, anchored enrichments, and other methods (reviewed in Funk, Zamudio, & Crawford, 2018). Genome‐wide data have improved species delimitation in amphibians, but its applications are much farther ranging and have immediate conservation implications. For instance, Pabijan et al. (2017) have shown that the well‐studied smooth newt complex of Europe contains eight different evolutionary lineages. Coalescent‐based demographic modeling revealed that four southern lineages have been genetically isolated for a substantial amount of time and deserve species status. These species harbor a significant amount of the phylogenetic and morphological diversity of the complex and have rather restricted distributions; a revision of their conservation status is therefore timely.

The new standard in pathogen detection and quantification (particularly *Bd* and *Bsal* spp., Ranavirus) in amphibians is quantitative PCR (qPCR) which has eliminated the need for laborious and unreliable histological examinations of amphibian skin (Boyle, Boyle, Olsen, Morgan, & Hyatt, 2004). This technique is being applied to an expanding assortment of pathogen species (Karwacki, Atkinson, Ossiboff, & Savage, 2018). Creative solutions using new techniques have been suggested for combating cane toad expansion; for instance, Tingley et al. (2017) floated the idea of using CRISPR/Cas9‐mediated targeted mutagenesis to knock out the toxin production pathway in toads and then use gene drive to spread the mutant in populations.

## 3 MIGRATION

### 3.1 Connectivity

Habitat fragmentation is a major cause of global biodiversity loss (Haddad et al., 2015). At the genetic level, fragmentation is associated with reduced gene flow, increased inbreeding, loss of diversity within populations, increased differentiation among populations, and elevated risk of extinction (Frankham, 2005). Understanding how changes in landscape composition and configuration affect intraspecific genetic variation and population viability is, however, an extremely challenging task, mainly due to the highly dynamic nature of landscapes (Epps & Keyghobadi, 2015; Richardson, Brady, Wang, & Spear, 2016). Nonetheless, studies at landscape genetic scales are widely recognized for their conservation potential (McCartney‐Melstad & Shaffer, 2015; Richardson et al., 2016) and aid conservation in three main ways (Bolliger, Lander, & Balkenhol, 2014): (a) by providing baseline information on dispersal and movement patterns; (b) advising conservation strategies; and (c) evaluating the effectiveness of conservation measures.

Strong links between landscapes and population connectivity in amphibians result from their relatively low mobility and limited ecological versatility, with most species showing pronounced phylogeographic and population genetic structure (Vences & Wake, 2007; Zeisset & Beebee, 2008), while some may function as classic metapopulations (Smith & Green, 2005), especially aquatic‐breeding species (e.g., Heard, Scroggie, & Malone, 2012). Regular extinction of local populations and dependence on recolonization make fragmentation a serious threat to the persistence of such species (Cushman, 2006; Rivera‐Ortíz, Aguilar, Arizmendi, Quesada, & Oyama, 2015). Recently, a summary of 42 amphibian landscape genetic studies confirmed that anthropogenic landscape features (e.g., urban areas, roads, and agricultural fields) had an overall negative effect on genetic connectivity (Cayuela et al., 2018), in agreement with studies at phylogeographic scales (see Section 8). Furthermore, Cayuela et al. (2018) identified other general patterns, such as the negative relationship between topographic complexity and gene flow or the importance of forested areas and networks of aquatic habitat for connectivity. Nonetheless, the applicability of these findings in conservation is limited because connectivity patterns vary widely across taxa and landscapes. Recent comparative landscape genetic studies on amphibians revealed contrasting connectivity patterns at all levels of evolutionary distinctiveness: urodeles versus anurans (Gutiérrez‐Rodríguez, Gonçalves, Civantos, & Martínez‐Solano, 2017), closely related frog species (Robertson et al., 2018), and even at intraspecific levels (Lourenço, Gonçalves, Carvalho, Wang, & Velo‐Antón, 2019). This finding has stimulated interest in multispecies inference and predictive frameworks, such as trait‐based approaches (Mims, Kirk, Lytle, & Olden, 2018). To give an example, in a species‐rich tropical anuran assemblage in Madagascar, small‐bodied species are poorer dispersers and thus show lower connectivity and stronger genetic structure over landscapes (Pabijan, Wollenberg, & Vences, 2012). Conservation measures maintaining or promoting population connectivity in the smaller species should safeguard most other species in this community. This approach has considerable conservation potential, especially given the growing availability of amphibian trait data (Mims et al., 2018), but it should be kept in mind that the relationships between traits and population connectivity will likely vary among regions and species assemblages.

The potential of genetic tools to advance connectivity research and aid conservation has long been recognized, but direct applications in amphibian conservation are still rare. Genetic evidence has linked pond network density with increased population connectivity in, for example, *H. arborea* (Angelone & Holderegger, 2009), *Ambystoma macrodactylum* (Savage, Fremier, & Bradley Shaffer, 2010), and *L. sylvaticus* (Coster, Babbitt, & Kovach, 2015). Landscape genetic studies following pond restoration include an assessment of European tree frog populations in Switzerland (Angelone & Holderegger, 2009) and palmate newt (*Lissotriton helveticus*) populations in France (Isselin‐Nondedeu et al., 2017). Both studies confirmed the restoration of ponds in a stepping‐stone manner as an effective measure to re‐establish connectivity among populations. Further genetic surveys are necessary to better understand the effects of such strategies on long‐term patterns of population genetic diversity and viability.

An important factor that is expected to further empower connectivity research and, by extension, practical conservation strategies is increased resolution of molecular markers. A recent study reassessing connectivity in the New York‐endangered eastern tiger salamanders (*Ambystoma tigrinum*) exemplifies the power of genome‐wide data in detecting effects of anthropogenic fragmentation (McCartney‐Melstad, Vu, & Shaffer, 2018). Using thousands of nuclear SNPs, the authors detected restricted connectivity between ponds separated by major roads, in stark contrast with a previous study based on microsatellites that apparently lacked power.

### 3.2 Range expansion

Although global change has had detrimental effects on most amphibians, a handful of generalist species have thrived in its wake by extending their ranges into formerly unoccupied areas. It is useful to differentiate between range expansion as the development of new population foci in the proximity of the native range of a species, and the expansion of introduced alien or invasive species far beyond their natural ranges (e.g., Zeisset & Hoogesteger, 2018). Although there are many parallels between the two, there are also differences (Moran & Alexander, 2014). Most notably, the establishment of an alien involves the removal of a species from its evolutionary context and places it within an entirely novel environment (see Section 6). In comparison, native populations only rarely undergo abrupt and severe environmental change and may also benefit from higher *Ne*, conductive toward adapting to new circumstances. Recent range expansions have been studied in the context of climate change in high‐altitude populations. Bosch et al. (2018) report expansions for three species into newly available habitat in the Peñalara region of Spain, and altitudinal expansion of three amphibian species in the Andes has been linked to recent deglaciation (Seimon et al., 2007). Habitat conversion has been implicated in the expansion of several generalist amphibian species, for example, *Hyperolius* in South Africa (Davies, Clusella‐Trullas, Hui, & McGeoch, 2013) and *Polypedates leucomystax* in South‐East Asia (Brown et al., 2010). The evolutionary implications of climate‐ or landscape‐related, recent range shifts in amphibians have not been explored thoroughly. However, an ongoing expansion of plains spadefoot toads (*Spea bombifrons*) into similar habitat (grassland) does not seem to decrease population connectivity or genetic diversity (measured as microsatellite variation) in the new populations; in contrast, expansion into novel habitat (desert) resulted in strong population differentiation, likely the result of bottlenecks and lack of connectivity (Pierce, Gutierrez, Rice, & Pfennig, 2017).

### 3.3 Implications for conservation

Genetic evidence clearly shows that human‐induced habitat fragmentation is the main factor behind the loss of genetic connectivity, leading to genetic erosion (see Section 2) and thus compromising the long‐term viability of amphibian populations. The process is predicted to accelerate through synergistic interactions with global climate change (Mantyka‐Pringle et al., 2015). European amphibians illustrate this problem, with numerous species expected to expand their ranges in response to climate change (Araújo, Thuiller, & Pearson, 2006). However, if dispersal and range expansion are constrained by habitat loss and fragmentation, amphibian populations will necessarily depend on their capacity for rapid adaptation for survival in the long term (see Section 4). On a more positive note, land cover can potentially buffer the negative effects of climate change because of its greater influence on patterns of connectivity among populations compared to climate, at least at local scales. Taking these factors into consideration, protecting the existing primary habitat patches and securing or increasing their quality should be the default conservation strategy (Ralls et al., 2018). However, the negative effects of increased connectivity (e.g., maladaptive gene flow, spread of disease and invasive species) should also be considered. For instance, among population gene flow has the potential to erase locally evolved, disease‐resistant genotypes in isolated populations of *Lithobates yavapaiensis* (Savage, Becker, & Zamudio, 2015). In this case, effective management should encompass activities aimed at preserving genetic diversity within populations (e.g., increasing effective population size) rather than promoting connectivity. Keeping in mind both the benefits and potential pitfalls of gene flow in a quickly changing world, we advocate considering the available genetic evidence directly linking landscape features and connectivity patterns in specific areas (mostly at local scales) when designing conservation strategies (Bolliger et al., 2014; Grant, Muths, Schmidt, & Petrovan, 2019).

The translocation of individuals is an important tool in the conservation of small, inbred populations through demographic or genetic restoration. Usually, nearby locations are chosen as donor sites because it is thought that individuals from distant populations will fare worse due to local adaptation and increased risk of outbreeding depression (Frankham et al., 2011). In a translocation experiment spanning nearly four decades, Zeisset and Beebee (2013) showed that repeated translocations of common toads (*Bufo bufo*) from nearby donor populations consistently failed to establish at a receptor site, whereas a single translocation from a distant site with different environmental characteristics became established and eventually thrived. The authors suggest that success was determined by the large size of the distant donor population, and by its higher mean fitness or more adaptive variation than the local but smaller donor sites. After 10 years, the receptor population retained allele frequency distributions at microsatellite loci similar to the donor site, but intriguingly MHC allele frequencies shifted to resemble local ones. This study implies that the risks of losing local adaptation can be offset if the introduced individuals originate from large populations because of a strong correlation between population size and fitness or adaptive variation in amphibians. Moreover, local selection acting on some loci of the genome can be very rapid (~3 toad generations), and these loci may often be involved in disease resistance. At a practical level, this study adds weight to the suggestion that the release of many individuals (>1,000) increases the chances of success of a translocation project (Germano & Bishop, 2009). Obtaining large numbers of animals for translocation by removal of eggs or larvae is entirely feasible for many species of aquatic‐breeding amphibians. Targeted or assisted gene flow involves moving individuals with advantageous traits to imperiled populations in an effort to increase viability (Kelly & Phillips, 2016). While promising, this approach has caveats including unpredictability and the potential for undesirable side effects such as the loss of genome‐wide genetic variation and adaptive potential. Management and decisions should carefully balance these liabilities against the risk of local extinction without intervention (O’Donnell, Drost, & Mock, 2017).

## 4 SELECTION

Literature abounds in examples of rapid phenotypic response to biotic and abiotic environmental factors, often of anthropogenic origin (Hoffmann & Sgro, 2011). Phenotypic shifts may not necessarily involve genetic changes, but can also be due to phenotypic plasticity, and distinguishing between the two is challenging (Merilä & Hendry, 2014). Only adaptation can prevent extinction in species unable to track suitable environment via range shifts (the concept of “evolutionary rescue,” reviewed in Bell (2017)). However, phenotypic plasticity and other nongenetic mechanisms may “buy time” and provide an opportunity for evolutionary rescue. Such nongenetic mechanisms may thus set the stage for adaptation (but also prevent it, see Ghalambor, McKay, Carroll, & Reznick (2007)) and also contribute to adaptation more directly, through genetic assimilation (Ehrenreich & Pfennig, 2015). Finally, plasticity itself can evolve. Because plastic responses of amphibians to environmental changes have recently been reviewed (Levis & Pfennig, 2019; Urban, Richardson, & Freidenfelds, 2014), we do not cover them here. Instead, we focus on genuine adaptive response and discuss two seemingly disparate, but closely linked issues: (a) whether the adaptive potential of amphibians is sufficient or can be increased to the point that it becomes relevant for conservation and (b) whether conservation interventions can prevent unwanted evolution.

### 4.1 The extent and rate of rapid adaptation in amphibians

A substantial fraction of the rapid phenotypic response to climate change in amphibians involves adaptive genetic changes (estimated proportion of genetic responses was 0.65, Urban et al. (2014)). This, and examples in Table 1, indicate considerable potential for rapid adaptation in amphibians. Other spectacular examples of fast phenotypic response also implicate genetic effects (e.g., Halfwerk et al., 2019; Vimercati, Davies, & Measey, 2018).

**Table 1:** Examples of recent or contemporary adaptation in amphibians

Taxon	Adaptation to	Response	Plasticity involved?	Evidence for genetic change	References
*Ambystoma maculatum*	Proximity of breeding site to roads	Environment‐dependent survival	ND (maternal effects cannot be excluded)	Genotype‐by‐environment interaction in a reciprocal transplant experiment	Brady (2012)
*Plethodon cinereus*	Habitat (forest cover) and temperature change	Proportion of two discrete color morphs differs between habitats	No	Color morph is genetically based	Cosentino, Moore, Karraker, Ouellet, & Gibbs (2017), but see Evans, Forester, Jockusch, & Urban (2018)
*Taricha granulosa*	Road deicing salts	Adaptive potential for salinity tolerance	ND	Variance in tolerance among families	Hopkins, French, & Brodie (2013)
*Lithobates sylvaticus*	Temperature change	In <40 years, thermal tolerance, preference, and temperature‐specific development rate have changed	No	Genotype‐by‐environment interaction in a reciprocal transplant experiment	Freidenburg & Skelly (2004); Skelly & Freidenburg (2000)
*Lithobates sylvaticus*	Insecticide	Higher constitutive insecticide tolerance close to agriculture, but higher induced tolerance far from agriculture	Yes	Differences in tolerance between populations in a gradient of distance from agriculture; pattern consistent with genetic assimilation	Hua et al. (2015)
*Lithobates sylvaticus*	Predation from *Ambystoma opacum* (predator range expands due to climate change)	Morphology, developmental rate, and behavior changed in response to predator presence	Yes	Genotype‐by‐environment interaction in a reciprocal transplant experiment	Urban et al. (2017)
*Lithobates sylvaticus*	Anthropogenic noise	Decreased physiological response to noise in populations exposed to anthropogenic noise	ND	Population‐level differences in response	Tennessen et al. (2018)
*Lithobates sylvaticus*	Urbanization	Directional changes in allele frequencies	ND	Genome‐wide association study supported an association between environment type and F_ST_ outlier loci identified in urban–rural comparison	Homola et al. (2019)
*Lithobates yavapaiensis*	*Bd* infection	Change in MHC allele frequencies	No	Signatures of ongoing positive selection on MHC alleles and supertypes in field and laboratory studies	Savage & Zamudio (2016)
*Rana arvalis*	Acid stress tolerance	Higher acid tolerance in populations exposed to acid conditions since the 1900s	Yes (maternal effect on survival under acid stress)	In reciprocal crosses between frogs from acid‐exposed and nonexposed populations, genetic effects found in development and growth	Räsanen, Laurila, & Merilä (2003)
*Rana temporaria*	Different pool‐drying patterns	Different growth rates and development rates caused probably by different foraging efforts	Yes	Support for a model assuming selection on standing genetic variation	Lind & Johansson (2011)
*Rhinella marina*	Dispersal opportunity	Dispersal abilities increased at the invasion front	ND	Heritable variation within and differences between populations	Phillips et al. (2010)
*Rhinella marina*	Low temperature (following invasion of new area)	Populations from cooler area have lower critical thermal minima	Yes	Persistent differences between populations after acclimation	Mittan & Zamudio (2019)

Abbreviations: *Bd*, *Batrachochytrium dendrobatidis*; ND, not determined.

A leading amphibian example for rapid adaptation to temperature change is provided by studies on the wood frog demonstrating shifts in thermal tolerance and preference as well as temperature‐specific developmental rate (Freidenburg & Skelly, 2004; Skelly & Freidenburg, 2000). Modeling by Skelly et al. (2007) suggested rapid evolution of critical thermal maxima, with estimated change of 3.2°C in 50 years, potentially mitigating the effects of predicted climate warming. However, this model assumes no loss of relevant variation during adaptation and ignores accompanying environmental change, and may therefore be too optimistic. Furthermore, the predicted rates of climate change probably dramatically exceed past rates of climatic niche evolution in vertebrates, including amphibians (Quintero & Wiens, 2013). Further examples document swift adaptation to, for example, novel predators, urbanization, insecticides, or contaminants (Table 1).

Of particular interest, given the threat it currently poses for amphibians worldwide, is the adaptive response to invasive fungal pathogens *Batrachochytrium dendrobatidis* (*Bd*) and *B. salamandrivorans* (*Bsal*). The adaptive potential to overcome the pathogen has been demonstrated for some amphibian populations (Kosch et al., 2019; Palomar, Bosch, & Cano, 2016). Changes in MHC allele frequencies (Savage & Zamudio, 2016) and other components of the immune system (Voyles et al., 2018) have been documented in response to *Bd* (see Section 7). Amphibian species often harbor considerable MHC variation providing raw material for adaptation (e.g., Fijarczyk, Dudek, Niedzicka, & Babik, 2018), which can, however, be rapidly lost, for example, following range expansion (Wielstra, Babik, & Arntzen, 2015). It is also known that particular sequence motifs, detected in multiple species, may affect susceptibility to *Bd* (Bataille et al., 2015). These two observations suggest that direct interventions promoting the spread of resistant alleles, such as selective breeding and targeted gene flow, may be conservation measures of immediate effect.

### 4.2 Preventing unwanted adaptation

The importance of ex situ amphibian conservation through captive breeding programs (CBP) is likely to increase (http://www.amphibianark.org/), even though captive assurance programs outnumber programs aimed at reintroductions (Harding, Griffiths, & Pavajeau, 2016). A major issue specific to CBP is adaptation to captivity, which can occur even in a single generation (Christie, Marine, French, & Blouin, 2012), and is detrimental to fitness in the wild (Frankham, 2008). Although origin of donor animals (wild, captive, or a combination) did not seem to affect translocation success (Germano & Bishop, 2009), only a small number of studies have looked into the issue. Indeed, a study on the Mallorcan midwife toad (*Alytes muletensis*) found that more than eight generations in captivity negatively affected not only genetic variation, but also a trait clearly related to fitness—induced antipredator defense (Kraaijeveld‐Smit, Griffiths, Moore, & Beebee, 2006). A recent modeling study revealed that even a small amount of adaptation to captivity can have a long‐term detrimental effect on natural populations supplemented with captive stock, with stronger consequences predicted for species with shorter life spans and higher rates of population replacement (Willoughby & Christie, 2019). Adaptation to captivity could be mitigated by applying the obvious but oftentimes impractical strategies of minimizing time spent in captivity or by ensuring that husbandry conditions are similar to those in the wild. The prospects of applying cryopreservation as an alternative for preventing unwanted evolution are unclear (Silla & Byrne, 2019). Another available strategy entails eliminating selection between families through equalization of family sizes (reviewed in Frankham, 2008). A further promising approach, supported by experimental evidence, is fragmentation, that is, the maintenance of several subpopulations in captivity and mixing before reintroduction. This slows down adaptation and effectively maintains variation and fitness at the level of the entire species/colony (Frankham, 2008). The effectiveness of this approach has not been examined in amphibians.

### 4.3 Implications for conservation

It remains largely unknown whether the rate and magnitude of adaptation in amphibians will be sufficient to prevent declines and extinction given the expected scale of environmental change in the coming decades. The prospects for successful adaptation are best in widespread species which could thus be subject to proactive conservation measures (Sterrett et al., 2019) but at the same time are, understandably, of lower conservation priority. Priority species often exhibit characteristics (i.e., small population size, depleted variation, large distance from fitness optimum) that reduce the likelihood of evolutionary rescue (Bell, 2017; Hoffmann et al., 2017). The scale of reduction of adaptive potential in endangered amphibians is currently unknown. However, there are reasons for moderate optimism regarding the prospect of adaptation to pathogen assault (Christie & Searle, 2018; Kosch et al., 2019; Voyles et al., 2018). Even threatened species often harbor the necessary genetic variation. Selective breeding, translocations that promote gene flow, and even interspecific introgression may be effective in some situations (see Section 5), while the risks of such interventions may be overestimated (Frankham et al., 2011). Conservation actions that increase fitness by reducing the mismatch between phenotype and environment, for example, the establishment of thermal refugia (see Section 7), are also worthy of further exploration, as they may reduce the demographic cost of selection that could otherwise lead to extinction. Finally, although the adaptive potential of amphibians in the context of global change may be limited, it is nevertheless important to consider the effect of contemporary evolution in projections of the future loss of amphibian biodiversity. Otherwise, the predicted loss is likely to be overestimated (Razgour et al., 2019).

## 5 HYBRIDIZATION

Hybridization, defined as mating between genetically distinct populations or species, can have a variety of evolutionary outcomes. It can cause the breakdown of reproductive barriers and fusion of lineages or, conversely, strengthen reproductive isolation and complete the speciation process. Hybridization can fuel adaptive radiations by providing ample genetic variation. From the conservation perspective however, the most important are shorter‐term (several tens of generations) consequences of hybridization and we focus on them here.

### 5.1 Hybridization as a challenge for conservation

Hybridization can disproportionately affect species of conservation concern, sometimes driving them to extinction (Todesco et al., 2016). Low hybrid fitness may cause demographic swamping, that is, when population growth rates of parental species fall due to wasted reproductive effort, leading to extinction. For instance, human‐mediated hybridization between the Gulf Coast toad (*Incilius nebulifer*) and Fowler's toad (*Anaxyrus fowleri*) has contributed to a decline of the latter due to strong postzygotic isolation (Vogel & Johnson, 2008). Hybrids of *Lithobates blairi* and *L. sphenocephalus* may cope less efficiently with emergent pathogens (Parris, 2004).

If hybrids are fit and population growth rates exceed replacement rates, then the parental species may be replaced by hybrids, a process referred to as genetic swamping (Todesco et al., 2016). Genetic swamping is often observed when a widespread species hybridizes with a rare, geographically restricted congener. For instance, hybridization between the Florida bog frog (*Lithobates okaloosae*) and the green frog (*L. clamitans*) puts into question the distinctiveness of the former (Austin, Gorman, Bishop, & Moler, 2011). Genetic swamping through human‐mediated introgressive hybridization between local species and exotic relatives is an important aspect of biological invasions. Hybridization between invasive barred tiger salamander *Ambystoma mavortium* and California tiger salamander *A. californiense* threatens the latter due to higher hybrid survival rates (McCartney‐Melstad & Shaffer, 2015). In Switzerland and the Netherlands, massive introgression from invasive *Triturus carnifex* resulted in local pollution of the genome of threatened *T. cristatus* (Dufresnes et al., 2016; Meilink, Arntzen, van Delft, & Wielstra, 2015). In extreme cases, hybridization can result in the formation of a population comprised entirely of hybrids (hybrid swarm) and, thus, the loss of a pure parental species—this appears to be the fate of most Swiss populations of the pool frog *Pelophylax lessonae* following invasion by the Italian pool frog (*P. bergeri*) (Dufresnes et al., 2017). *Pelophylax* frogs seem to be particularly prone to lineage fusion after introduction: Molecular sequence analysis has revealed numerous cases of cryptic translocations of several exotic lineages in Europe. The extent and consequences of the resulting introgression events are unclear, but may dramatically affect the viability of native hybridogenetic populations through demographic or genetic swamping (Dufresnes & Dubey, 2020).

From a broader perspective, hybrids can also negatively impact other amphibian species. For example, hybrid tiger salamander larvae dramatically reduced the survival of two native amphibians (Ryan, Johnson, & Fitzpatrick, 2009). Hybridization and subsequent recombination between strains can generate new, highly virulent pathogen genotypes, as in the case of two *Bd* lineages in Brazil's Atlantic Forest. The virulence of the new hybrid lineage exceeded that of both parents in some host species, suggesting that novelty arising from hybridization between *Bd* strains is of conservation concern (Greenspan et al., 2018).

### 5.2 Hybridization as an opportunity for conservation

Hybridization may also facilitate conservation (Hamilton & Miller, 2016). First, hybridizing species may have access to a larger pool of genetic variation than species evolving independently (Hedrick, 2013), which may increase their adaptive potential. Introgressive hybridization from the Mexican spadefoot toads (*Spea multiplicata*) may have facilitated the expansion of plains spadefoot toads into novel habitat in the southwestern United States (Pierce et al., 2017). Beneficial alleles can cross species barriers relatively easily even at advanced stages of speciation, when selection against hybrids is strong and the risk of genetic swamping is low. For example, the highly diverged newts *Lissotriton. vulgaris* and *L. montandoni* demonstrate massive, most likely adaptive introgression of MHC (Dudek, Gaczorek, Zieliński, & Babik, 2019) and possibly other immune genes (Fijarczyk et al., 2018).

Second, extensive and asymmetric mtDNA introgression may involve the replacement of mutationally loaded mtDNA by molecules of lesser load, preventing mutational meltdown in the recipient species (Hill, 2019). Newts from the *L. vulgaris* complex (Pabijan et al., 2017) are a possible example. However, interspecific crosses are rarely used for genetic rescue due to concerns about outbreeding depression (Kovach, Luikart, Lowe, Boyer, & Muhlfeld, 2016). Nevertheless, in cases of severe inbreeding depression, human‐assisted hybridization should be seriously considered as a last‐ditch resort for genetic rescue, even though such interventions are reaching the limits of what conservation is.

Finally, hybridization may lead to reinforcement of prezygotic isolation. In the hybrid zone between North American upland chorus frog (*Pseudacris feriarum*) and southern chorus frog (*P. nigrita*), reinforcement has led to the evolution of both female preferences and male signals, resulting in increased prezygotic isolation countering maladaptive hybridization (Lemmon & Lemmon, 2010).

#### 5.2.1 Implications for conservation

Hybridization is of conservation concern because of the risk of demographic and genetic swamping; however, it is also a potential source of adaptive genetic variation. Predicting which is more likely following secondary contact has management value. Although the negative effects of hybridization have been emphasized, only a few studies have assessed the fitness effects of introgressed alleles. This information is crucial for an understanding of the consequences of hybridization in maintaining species distinctiveness and adaptive potential (Kovach et al., 2016), and also its effectiveness as a tool used in conservation interventions.

Growing trade and climate change will likely cause further amphibian range shifts and invasions. Therefore, a global increase in hybridization frequency is expected. However, the legal status of genetically admixed individuals remains largely unresolved. Hence, coherent policy on hybrids and hybridization is urgently needed. In our opinion, naturally occurring hybrids are part of the evolutionary process and as repositories of their parental genomes should be subject to the same conservation policy as parental species. Regarding the protection status of hybrids resulting from human‐mediated hybridization, we argue that decisions should be made on a case‐to‐case basis to maximize the retention of genetic diversity, distinctiveness, and fitness of species involved (Wayne & Shaffer, 2016). Hybridization is still an underexplored and underappreciated source of genetic variation which can be utilized for genetic rescue of species when there is no possibility of intraspecific population crosses. As the amphibian conservation crisis worsens, the propagation of genetic variation via human‐mediated hybridization may become a valuable part of the amphibian conservationist toolbox.

## 6 EVOLUTIONARY EFFECTS OF INVASIVE AMPHIBIANS

The intentional or inadvertent introduction of alien amphibian species has occurred hundreds of times across the globe (Kraus, 2008). Of the dozens of species that have established populations far outside of their native ranges, seven (in descending impact: *Rhinella marina*, *Xenopus laevis*, *Duttaphrynus melanostictus*, *Ambystoma tigrinum*, *Lithobates catesbeianus*, *Eleutherodactylus coqui,* and *Osteopilus septentrionalis*) have undisputed and significant ecological impacts and have generated the most scientific interest (Measey et al., 2016). Some of the world's most highly invasive amphibian species (e.g., *Xenopus laevis, Rhinella marina,* and *Lithobates catesbeianus*) are also subclinical carriers of deadly amphibian pathogens and aid in their spread and maintenance in the environment (Fisher & Garner, 2007). The consequences of amphibian introductions include increased predation, competition, hybridization (see Section 5), and others, as well as socio‐economic impacts to human well‐being and food production (Kraus, 2008, 2015; Measey et al., 2016). Many of these changes may have evolutionary consequences in both the invading species and the impacted native species (Shine, 2012).

One common finding is that successful establishment of invasive amphibians is not contingent on the genetic variation present in the founding population: Most amphibian invaders have low genetic diversity yet exhibit spatial and demographic growth (e.g., Peacock, Beard, O’Neill, Kirchoff, & Peters, 2009). Moreover, introduced populations may themselves be sources of secondary introductions (Heinicke, Diaz, & Hedges, 2011).

Much of the information on evolutionary repercussions in invading species comes from comprehensive analyses of the cane toad (*Rhinella marina*), the natural range of which encompasses Central and tropical South America. This species has been intentionally introduced into other tropical regions, most notably into northern Australia and many Pacific islands. All life stages of this toad produce toxic substances, and lethal ingestion is the primary pathway of direct impact on native species. In Australia, the range of this species has expanded rapidly over the last eight decades (Urban, Phillips, Skelly, & Shine, 2008). A reduction in genetic diversity after the introduction does not seem to have affected ecologically relevant traits in cane toads (Selechnik et al., 2019); instead, the up‐regulation of a suite of genes related to metabolism, energetics, and immune function may hold the key to increased invasiveness in this species (Rollins, Richardson, & Shine, 2015). The spread of toads in Australia has resulted in the appearance of a range‐expansion phenotype manifested in dispersal‐prone behavior, faster growth rates, longer leg lengths, and greater stamina in toads at the invasion front (Lindström, Brown, Sisson, Phillips, & Shine, 2013; Phillips, Brown, Webb, & Shine, 2006). Common‐garden experiments suggest that many of these traits have a genetic basis and are potentially adaptive (Gruber, Brown, Whiting, & Shine, 2017; Phillips, Brown, & Shine, 2010). However, there is also evidence that faster dispersal in toads at the invasion front is a result of the spatial sorting of alleles determining higher mobility: Such alleles will inevitably accumulate at the range margin leading to assortative mating by dispersal in toads (Phillips et al., 2006; Shine, Brown, & Phillips, 2011). Thus, adaptive evolution in both life‐history and dispersal traits, spatial sorting due to nonequilibrium demographics, and epigenetic mechanisms have all contributed to the invasiveness of this species (Perkins, Phillips, Baskett, & Hastings, 2013). In line with the cane toad example, a recent study documented longer limbs and greater stamina in peripheral populations of the invasive African clawed frog (*Xenopus laevis*) in France (Louppe, Courant, & Herrel, 2017), albeit at a much smaller spatial scale.

In another toad example (Vimercati et al., 2018; Vimercati, Davies, & Measey, 2019), an invasive population of the guttural toad (*Sclerophrys gutturalis*) in South Africa has evolved a suite of behavioral, phenotypic, and reproductive traits that allow it to better cope with the drier conditions in its new range. However, it is not yet possible to distinguish whether these shifts represent local adaptation or phenotypic plasticity and thus should be viewed as responses lowering phenotypic mismatches between invasive species and their novel environments.

A recent meta‐analysis found that alien species of a wide range of taxonomic groups (but especially fish and crayfish) have detrimental effects on native amphibian species (Nunes et al., 2019). However, native amphibian populations may also respond adaptively, reducing the invaders’ impact or exploiting the novel opportunity it provides (reviewed in Shine, 2012 and Table 1).

### 6.1 Implications for conservation

Identifying the provenance of extralimital amphibian populations is important for prioritizing conservation actions (do we eradicate or protect?) but can be tricky. Importantly, the contrasting population genetic hallmarks of expansions in human‐mediated versus landscape‐ or climate‐mediated range expansions can be put to work for this purpose. For instance, Tolley, Davies, and Chown (2008) pitted jump dispersal versus diffusion population genetic signatures to resolve the status of extralimital populations of the painted reed frog (*Hyperolius marmoratus*) in southern South Africa, finding that all new populations were most likely the result of human transport and thus should be considered alien and potentially invasive.

Research on several species showed that the phenotypes of invasive amphibians may undergo rapid optimization in their new habitats which, coupled with increased dispersal ability, may render invasive populations unmanageable. Unfortunately, after establishment, alien‐species naturalizations are usually irreversible. Novel evolutionary measures against invasive amphibian populations have been proposed. One as yet untested idea to counter the hyperdispersive toads leading the cane toad expansion in northwestern Australia is to introduce individuals of lower dispersal capacity (from established populations) to the invasion front. It is thought that this “genetic backburning” strategy (Tingley et al., 2017) could stop the toads from dispersing across dry terrain and curb the colonization of areas of more amiable climate. Another idea is to facilitate the spread of natives adapted to invasion. Rapid adaptation in the form of behavioral avoidance of toads as prey suggests the use of targeted gene flow (Kelly & Phillips, 2016) to introduce toad avoidance into toad naïve populations of would‐be predators.

## 7 COEVOLUTION WITH PATHOGENS

Pathogens and hosts with shared evolutionary history have often coevolved to stable coexistence. Environmental stressors, such as climate change, pollution, and alien species, can tip the balance in favor of pathogens or introduce new pathogens to naïve hosts (Box 1; Blaustein & Kiesecker, 2002; Blaustein et al., 2018). Furthermore, the presence of multiple stressors interacting with amphibian diseases complicates the identification of disease‐specific effects and predictions relevant for conservation. In this section, we review evolutionary aspects of amphibian diseases: susceptibility, evolutionary consequences, coinfections, and management strategies.

### 7.1 Susceptibility to disease: the sum of elements

Many aspects of life and evolutionary histories of hosts and pathogens shape the evolution of susceptibility to disease, leading to enormous variation among individuals, populations, and species (Gervasi et al., 2017; Lips, 2016). Different strains of pathogens can differ in virulence, as described for ranavirus (Duffus et al., 2015) and *Bd* (O’Hanlon et al., 2018). Regarding hosts, three aspects should be considered. First, microbiota play an important role, since they may impede or facilitate pathogen infection (Jiménez & Sommer, 2017). Second, amphibian life‐history traits and behaviors may coevolve with pathogens. Genetic and phenotypic correlations were detected between larval period length and *Bd* load (Palomar et al., 2016) and body size affects the rate of ion loss and energetic demand during chytridiomycosis, making smaller individuals more vulnerable (Wu, Cramp, & Franklin, 2018). Likewise, elevated body temperatures decrease *Bd* infection probability and help to overcome *Bd* and ranavirus in some species (Rowley & Alford, 2013; Sauer et al., 2018; Sauer, Trejo, Hoverman, & Rohr, 2019). Hence, thermoregulatory behaviors as well as other behaviors mitigating infection risk or fitness cost may be subject to selection (Kelleher, Silla, & Byrne, 2018; Koprivnikar, Gibson, & Redfern, 2012). Finally, host immune response genes are major drivers of host–pathogen coevolution. Some MHC alleles participate in amphibian resistance against several bacteria, fungi, and viruses (see Section 4, Barribeau, Villinger, & Waldman, 2008; Bataille et al., 2015; Teacher, Garner, & Nichols, 2009a), and higher overall genetic diversity increases survival at the population level (e.g., Pearman & Garner, 2005). The dynamics of chytridiomycosis could be driven largely by host capacity for rapid adaptation, as there is little evidence of *Bd* evolving to reduce its virulence in the short term (Voyles et al., 2018)—if so, conservation management should focus on host adaptation. Environment also influences coevolution between hosts and pathogens. For instance, ephemeral aquatic environments have been linked to high susceptibility of some species to *Bd* (Gervasi et al., 2017), while permanent, lotic environments seem to increase infection prevalence and intensity for other species (Kriger & Hero, 2007). Furthermore, temperature‐dependent immunity in amphibians (e.g., Raffel, Rohr, Kiesecker, & Hudson, 2006) may promote the evolution of pathogen life‐history strategies that exploit periods of increased host susceptibility (Woodhams, Alford, Briggs, Johnson, & Rollins‐Smith, 2008). In sum, multiple factors affect the evolution of susceptibility, but an understanding of their individual contributions and, in particular, of their interactions is far from complete.

### 7.2 Evolutionary consequences of disease

Infectious disease can profoundly affect host evolution. Responses to other stressors, such as predation risk, might be compromised in infected individuals (Rae & Murray, 2019). Furthermore, genotype‐dependent fitness differences rapidly change the genetic composition of populations (reviewed in McKnight, Schwarzkopf, Alford, Bower, & Zenger, 2017): Diversity may decrease both overall, due to population crashes, and around genomic targets of selection. Selection imposed by disease prompts two nonexclusive responses: resistance defense, limiting pathogen burden, and tolerance strategy, reducing the fitness costs. These responses are expected to have different evolutionary consequences. Alleles conferring tolerance tend to fix if disease prevalence remains high, because they provide universal advantage in the presence of the pathogen and the allele is permanently beneficial. In contrast, loci conferring resistance are usually polymorphic because the selective advantage of particular resistance alleles tends to deteriorate under decreasing prevalence (Roy & Kirchner, 2000). For instance, many studies have documented how diseases drive the evolution and maintenance of MHC variation (reviewed in Spurgin & Richardson, 2010). These evolutionary consequences apply not only to alleles but also to behaviors, microbiota composition, life‐history traits, and other factors under selection by the disease. Which defense strategy is selected for by disease is context‐dependent. On the one hand, if selection favors a strategy that allows a host genotype to outperform competitors by creating a harsh parasitic environment, by maintaining the pathogen reservoir, then high tolerance and low resistance may evolve (Restif & Koella, 2004; Venesky, Raffel, McMahon, & Rohr, 2014). On the other hand, low rates of pathogen exposure and high pathogen virulence should favor resistance over tolerance (Restif & Koella, 2004). Interestingly, the strategy can even depend on amphibian host age and developmental stage. For instance, Pacific chorus frogs (*Pseudacris regilla*) are tolerant to trematode infection during larval development and resistant after metamorphosis (Johnson, Kellermanns, & Bowerman, 2011). Understanding the relative importance of host tolerance/resistance can have relevant connotations for amphibian conservation (Venesky et al., 2014) as *Bd*‐induced extinction dynamics are more sensitive to host resistance/tolerance than to *Bd* transmission (Wilber, Knapp, Toothman, & Briggs, 2017).

Gene flow patterns will also be modified by diseases due to changes in behavior or dispersal (Teacher, Garner, & Nichols, 2009b) and the reduction of population size, increasing fragmentation, and disconnection between patches (see Section 3). Population dynamics will be altered if mortality due to disease is conditional on life stage or sex, leading to biased age structure or sex ratio (Rosa et al., 2019; Scheele et al., 2019). Implications include destabilization of the population networks, decrease in population resilience, strong selection on some life‐history traits, and dependence on consistent and high recruitment (Scheele et al., 2019). Some species or populations will simply not be able to adapt because of limited genetic variation, trade‐offs, or physiological constraints, which may lead to extinction (see Sections 2 and 4). Finally, it is important to emphasize that host–pathogen dynamics are contingent on environmental factors (James et al., 2015; Savage et al., 2015).

### 7.3 Coinfection, the great unknown

Coinfection by two or more pathogens is commonly reported in amphibians (Olori et al., 2018; Warne, LaBumbard, LaGrange, Vredenburg, & Catenazzi, 2016), but its consequences for host–pathogen coevolution are poorly known. Correlations between some infections have been detected (Stutz et al., 2018), and hybridization between different pathogen lineages has been demonstrated (see Section 5). Coinfections may have diverse outcomes for host fitness. Some pathogens attenuate host immune response, opening the door to other pathogens. For instance, *Bd* produces toxins that impair lymphocyte proliferation and induce apoptosis in the amphibian skin (Fites et al., 2013) and in consequence may facilitate infection of the skin by the ectoparasite *Gyrodactylus jennyae* (Paetow, McLaughlin, Pauli, & Marcogliese, 2013). In contrast, cross‐reactive immunity or competence may reduce the effect of a second pathogen (Hoverman, Hoye, & Johnson, 2013). For example, prior infection with trematode parasites reduced ranavirus loads and increased survival (Wuerthner, Hua, & Hoverman, 2017). Unfortunately, this was not the case when animals that cleared *Bd* infection were infected with *Bsal* (Longo, Fleischer, & Lips, 2019). The understanding of processes such as competition between pathogens, cross‐reactive immunity, and immune suppression in amphibians would help to develop new conservation measures.

### 7.4 Implications for conservation

From antivirals, antifungals, and salt treatment to exposure to dead pathogens or high temperatures (Jiménez & Sommer, 2017; McMahon et al., 2014; Woodhams et al., 2011), conservation strategies have attempted to minimize the mismatch between host phenotype and fitness optimum, and therefore can be considered evolutionary in a broad sense. However, there are few examples of strategies explicitly considering evolutionary principles of pathogen–host coevolution and effective mitigation strategies in situ are still lacking. Although there is no general recipe, some interventions could be applied in the short term to prevent immediate population collapse, increasing the opportunity for adaptation. Some reports suggest that chytridiomycosis might be alleviated by bioaugmentation of locally occurring protective bacteria (Jiménez & Sommer, 2017; Woodhams et al., 2011) although the practical utility of this measure still needs to be demonstrated. Management approaches manipulating the environment to create climatic refuges from disease may be an option (Richards‐Zawacki, 2009; Scheele et al., 2019), for example, by reducing canopy cover within an aquatic environment to create warm sites above the tolerance limit of *Bd*, facilitating clearance in amphibian hosts native to warm environments (Hettyey et al., 2019), or increasing canopy cover creating cooler sites for clearance of amphibians adapted to cold environments (Sauer et al., 2019). Identification of the loci involved in resistance/tolerance could be a breakthrough in amphibian conservation. Assessment of susceptibility based on molecular markers could prioritize and focus conservation efforts (Bataille et al., 2015; Woodhams et al., 2011), and integration of genetic and environmental data may help in understanding the evolutionary response to disease (Horner, Hoffman, Tye, Hether, & Savage, 2017; Savage et al., 2015). Furthermore, the introduction of resistant or tolerant individuals into populations threatened by disease or as new population foci (the “recovery engine strategy” for *Bd* (Mendelson, Whitfield, & Sredl, 2019)) could facilitate adaptation and drive the re‐establishment of extirpated populations. This explicitly evolutionary approach is in urgent need of assessment—Joseph and Knapp (2018) provide one successful example in a high‐altitude population of *R. sierra*, while anecdotal data suggest that the method may work in tropical latitudes as well. Another as of yet untested approach entails the release of numerous surplus amphibians (e.g., at the tadpole stage) from CBP into suitable habitat in which *Bd* is present with the expectation that natural selection will eventually generate a tolerant or resistant variant (Lewis et al., 2019). Amphibian farming and trade are major contributors to the spread of chytridiomycosis and ranavirosis (Picco & Collins, 2008; Ribeiro et al., 2019). Hence, conservation efforts eradicating introduced species would help amphibian communities by reducing the probability of infection via decreasing reservoirs.

## 8 CONSERVATION OF MACROEVOLUTIONARY PROCESSES: MAINTAINING PHYLOGENETIC AND FUNCTIONAL DIVERSITY IN AMPHIBIAN COMMUNITIES

The in situ conservation of amphibians relies on the establishment of protected areas. For many species, site‐based protection may be the primary strategy to ensure their survival. With a limited budget and continuing habitat conversion across the globe, decisions on where to place new protected areas or expand existing ones are not a trivial matter. Nearly a quarter of amphibian species are not encompassed by any kind of protected area, despite a global increase in their extent (Nori et al., 2015). New research emphasizes that an evolutionary perspective is essential in the planning of protected areas for the preservation of amphibian biodiversity. Instead of using traditional measures such as species counts based on taxonomic categorization, conservationists are increasingly turning to phylogenetic measures (e.g., phylogenetic diversity and evolutionary distinctiveness) as standards used in conservation planning. These measures are based on the phylogenetic relationships of organisms and have two main advantages over traditional measures: They bypass the problem of defining species and can be used as a surrogate for evolutionary potential. The second is particularly crucial in conservation science since a loss of evolutionary potential may be more important than a loss of species per se. One such measure is evolutionary or genetic distinctiveness (Redding & Mooers, 2006) which gives greater value to species whose evolutionary history is not shared with many other species. Combined with an assessment of the level of threat of a species (typically IUCN categories), evolutionary distinctiveness can help to prioritize areas for protection and target species of special concern (Isaac, Redding, Meredith, & Safi, 2012). For instance, old lineages contribute more phylogenetic diversity than younger lineages and many are at risk of extinction, the top five most in need of conservation action being *Leiopelma*, *Andrias*, *Boulengerula*, *Nasikabatrachus,* and *Telmatobufo* (Isaac et al., 2012). Greenberg, Palen, Chan, Jetz, and Mooers (2018) used evolutionary distinctiveness to compare natural and disturbed habitats across the globe and showed that intact (tropical) forests “span the diversification continuum” in amphibians, encompassing both evolutionarily distinct lineages and clades that have undergone rapid diversification. These results emphasize that losing tropical forest will entail the loss of a substantial amount of amphibian evolutionary history, and thus provide one more reason why in situ conservation efforts should prioritize what remains of the world's tropical forests.

Another study that used phylogenetic diversity measures as a basis for conservation guidelines (Nowakowski, Frishkoff, Thompson, Smith, & Todd, 2018) showed that closely related amphibian species (at the level of genera or families) respond similarly to human‐induced habitat change. Most amphibians (ca. 80%), especially clades associated with pristine tropical rainforest such as *Pseudophilautus* (Rhacophoridae), *Craugastor* (Craugastoridae), *Pristimantis* (Strabomantidae), and the Neotropical salamanders *Bolitoglossa* (Plethodontidae), are being disproportionately affected by habitat conversion. These lineages are typically species‐rich and share some life‐history and ecological traits; for instance, many species have lost the free‐swimming larval stage, have small body sizes, and inhabit pristine rainforest. Their survival hinges on the protection of large swathes of rainforest in countries that are undergoing human population expansion and rapid development. Nowakowski et al. (2018) found that other clades (about 20% of amphibian species) seem pre‐adapted to novel conditions after habitat conversion and may even thrive; some notable examples include Asian grass frogs of the genus *Fejervarya* (Dicroglossidae) that inhabit rice paddies, *Dendropsophus* treefrogs (Hylidae) that live in deforested areas of the Neotropics, and some members of the large families Leptodactylidae, Ranidae, and Bufonidae.

Another area‐based conservation parameter is a community's functional diversity (Cadotte, Carscadden, & Mirotchnick, 2011). This approach assumes that the diversity of species traits in an assemblage in a predefined area is representative of species niche diversity. Functional diversity is thought to underlie a number of important ecosystem properties such as stability and resilience (Loreau & De Mazancourt, 2013) and therefore should be included in conservation planning. Functional diversity has been used in conjunction with phylogenetic and taxonomic diversity in an effort to optimize the planning of protected habitat for amphibians in the Brazilian Atlantic forest hotspot (e.g., Campos, Lourenço‐de‐Moraes, Llorente, & Solé, 2017). Habitat loss alters the functional diversity of amphibian communities by changing or reducing their functional complexity (Almeida‐Gomes, Vieira, Rocha, & Melo, 2019; Riemann, Ndriantsoa, Rödel, & Glos, 2017). Joint consideration of functional and phylogenetic diversity is essential, because it remains unclear to what extent phylogenetic relatedness determines similarity in the roles species play in ecosystems (Webb, Ackerly, McPeek, & Donoghue, 2002). Including these two measures of diversity allows combining ecological and evolutionary approaches into conservation decision‐making (Lourenço‐de‐Moraes et al., 2019).

### 8.1 Implications for conservation

These studies have several ramifications for amphibian conservation. First, expanding current protected areas, especially in tropical countries, is necessary if we want to preserve a large part of amphibian evolutionary history. Policymakers should target remnant forests as these will tend to encompass both evolutionarily distinct lineages and closely related species that have recently diversified. Phylogenetic distinctiveness and level of threat form the basis for constructing Evolutionarily Distinct and Globally Endangered species lists (EDGE lists), and one is now available for amphibians (Isaac et al., 2012). Second, since a large proportion of amphibian clades are present outside of protected areas, maintaining extensive agricultural management practices that provide ecological diversity can help maximize amphibian phylogenetic (and species) diversity in a human‐dominated landscape. Third, human‐induced changes in the environment have increased available habitat (and/or decreased competition) for some species (Nowakowski et al., 2018).

Can habitat restoration (the design of secondary communities) revive threatened amphibians, especially the evolutionary unique, specialist species? There is some evidence that this indeed may be the case. In a study examining the effects of habitat restoration in southern Mexico, Díaz‐García, Pineda, López‐Barrera, and Moreno (2017) showed that after 7 years, restoration areas adjacent to forest fragments contained a combination of cloud forest specialist as well as generalist species that came close to pristine sites in terms of species and functional diversity. Unfortunately, habitat restoration does not afford protection from threats such as climate change or infectious diseases. Ideally, protected areas should be large enough to sustain populations of target species and to act as sources of colonizers for smaller habitat patches in the landscape and should also envelope as much phylogenetic and functional diversity as possible.

## 9 CONCLUSIONS: AN EVOLUTIONARY PERSPECTIVE ON MANAGING ECOSYSTEMS FOR AMPHIBIANS

It is abundantly clear from our review that amphibian populations can evolve to accommodate new environmental situations. However, the evolutionary response may not be rapid enough to cope with the pace of current global change. Collectively, the field has amassed a substantial amount of information on evolutionary processes in amphibians and we have a reasonable idea of what to protect and how to prioritize protection in the case of limited resources. However, there is a dearth of studies that have attempted to implement evolutionary principles in or that are directly informative for amphibian conservation. Table 2 lists some fundamental research questions that could be pursued in this respect. Ideally, baseline information for each species on a conservationist's agenda could be obtained through detailed and time‐consuming studies. Under the current circumstances, however, a common sense (“quick and dirty”) approach may be a legitimate alternative. For instance, predictive frameworks linking genetic cohesion to functional traits of the amphibian community will probably succeed in determining the extent of necessary habitat restoration in a given area, without the need of conducting landscape genetic studies of each constituent species.

**Table 2:** In need of further study—evolutionary research questions of fundamental importance for amphibian conservation

Drift and inbreeding
Information on the effectiveness of genetic rescue More data on *Ne* of local populations and the mechanisms supporting relatively high *Ne*/*N* ratios
Migration
Landscape genetic studies aimed at evaluating the effectiveness of habitat restoration on population genetic cohesion Fragmentation and management of gene flow among captive colonies as a tool for minimizing adaptation to captivity and loss of variation.
Selection
Does adaptation to captivity affect translocation success? What are the short‐ and long‐term costs of diluting local adaptation through translocation?
Hybridization
Do the benefits of hybridization as a source of adaptive genetic variation typically exceed risks?
Evolutionary effects of invasive amphibians
Adaptation versus phenotypic plasticity versus spatial sorting—more experimental data needed to verify potential adaptive scenarios Can manipulation of phenotypes/genotypes at the expansion front impede the spread of invasive species?
Coevolution with pathogens
Identification of loci involved in resistance/tolerance Can the introduction of resistant or tolerant individuals into populations threatened by disease facilitate adaptation?
Conservation of macroevolutionary processes
Can habitat restoration revive threatened amphibians, especially the evolutionary unique, specialist species?

Can evolutionary principles aid in halting amphibian declines? Yes, by securing the adaptive potential of (declining) amphibian species, by optimizing habitat management to ensure landscape‐scale population dynamics, by reducing phenotype–environment mismatch, and by recognizing and spreading fitness‐increasing variants. A number of conservation tools have been proposed, including evolutionary approaches to captive breeding aimed at increasing *Ne* and minimizing unwanted adaptation, selective breeding, and targeted gene flow for specific traits or adaptive variants (e.g., disease resistance), translocations of more fit individuals to populations experiencing fitness declines, and interspecific hybridization as a source of adaptive variation. Few of these tools have actually been implemented in amphibian conservation, and all are in need of evaluation. We also raise awareness of the possibility that the implementation of evolutionary solutions to amphibian conservation may have ecological implications for the local communities. Most obviously, actions that directly modify habitat such as breeding site restoration (aimed at securing population connectivity) or establishment of thermal refugia (to mitigate disease) will likewise affect the local ecology. However, translocations of individuals or genes may also seriously and unpredictably alter local associations. Ideally, the potential conflict between an evolutionary solution for an imperiled amphibian species and the ecological outcome of the action should be acknowledged and factored into a conservation strategy.

Protecting the existing primary habitat patches and increasing their quality or extent is a direct conservation measure of immediate effect and is essential for many nongeneralist species. However, as we watch most pristine ecosystems, especially tropical forests, disappear, secondary habitat in disturbed areas or restored forest is becoming increasingly important for many amphibian species. An evolutionary perspective incorporating phylogenetic and functional diversity is essential in the planning of the secondary habitat, and management approaches manipulating the environment to create climatic refuges from disease could become valid approaches in a more homogenous landscape.

## CONFLICT OF INTEREST

None declared.

## Data Availability

This article does not contain data.

## References

[eva12940-bib-0001] Allentoft, M. E. , & O’Brien, J. (2010). Global amphibian declines, loss of genetic diversity and fitness: A review. Diversity, 2, 47–71.

[eva12940-bib-0002] Almeida‐Gomes, M. , Vieira, M. V. , Rocha, C. F. , & Melo, A. S. (2019). Habitat amount drives the functional diversity and nestedness of anuran communities in an Atlantic Forest fragmented landscape. Biotropica, 51, 874–884.

[eva12940-bib-0003] Álvarez, D. , Lourenço, A. , Oro, D. , & Velo‐Antón, G. (2015). Assessment of census (*N*) and effective population size (*Ne*) reveals consistency of Ne single‐sample estimators and a high *Ne*/*N* ratio in an urban and isolated population of fire salamanders. Conservation Genetics Resources, 7, 705–712.

[eva12940-bib-0004] Angelone, S. , & Holderegger, R. (2009). Population genetics suggests effectiveness of habitat connectivity measures for the European tree frog in Switzerland. Journal of Applied Ecology, 46, 879–887.

[eva12940-bib-0005] Araújo, M. B. , Thuiller, W. , & Pearson, R. G. (2006). Climate warming and the decline of amphibians and reptiles in Europe. Journal of Biogeography, 33, 1712–1728.

[eva12940-bib-0006] Austin, J. , Gorman, T. , Bishop, D. , & Moler, P. (2011). Genetic evidence of contemporary hybridization in one of North America's rarest anurans, the Florida bog frog. Animal Conservation, 14, 553–561.

[eva12940-bib-0007] Bálint, M. , Nowak, C. , Márton, O. , Pauls, S. U. , Wittwer, C. , Aramayo, J. L. , … Jansen, M. (2018). Accuracy, limitations and cost efficiency of eDNA‐based community survey in tropical frogs. Molecular Ecology Resources, 18, 1415–1426. 30155977 10.1111/1755-0998.12934

[eva12940-bib-0008] Barribeau, S. M. , Villinger, J. , & Waldman, B. (2008). Major histocompatibility complex based resistance to a common bacterial pathogen of amphibians. PLoS ONE, 3, e2692. 18629002 10.1371/journal.pone.0002692 PMC2443284

[eva12940-bib-0009] Bataille, A. , Cashins, S. D. , Grogan, L. , Skerratt, L. F. , Hunter, D. , McFadden, M. , … Harlow, P. S. (2015). Susceptibility of amphibians to chytridiomycosis is associated with MHC class II conformation. Proceedings of the Royal Society B: Biological Sciences, 282, 20143127. 10.1098/rspb.2014.3127 PMC4389617 25808889

[eva12940-bib-0010] Beauclerc, K. B. , Johnson, B. , & White, B. N. (2010). Genetic rescue of an inbred captive population of the critically endangered Puerto Rican crested toad (*Peltophryne lemur*) by mixing lineages. Conservation Genetics, 11, 21–32.

[eva12940-bib-0011] Beebee, T. (2005). Conservation genetics of amphibians. Heredity, 95, 423‐427. 16106261 10.1038/sj.hdy.6800736

[eva12940-bib-0012] Beebee, T. (2009). A comparison of single‐sample effective size estimators using empirical toad (*Bufo calamita*) population data: Genetic compensation and population size‐genetic diversity correlations. Molecular Ecology, 18, 4790–4797. 19863715 10.1111/j.1365-294X.2009.04398.x

[eva12940-bib-0013] Beebee, T. J. (2018). Genetic contributions to herpetofauna conservation in the British Isles. Herpetological Journal, 28, 51‐62.

[eva12940-bib-0014] Bell, G. (2017). Evolutionary rescue. Annual Review of Ecology, Evolution, and Systematics, 48, 605–627.

[eva12940-bib-0015] Blaustein, A. R. , & Bancroft, B. A. (2007). Amphibian population declines: Evolutionary considerations. BioScience, 57, 437–444.

[eva12940-bib-0016] Blaustein, A. R. , & Kiesecker, J. M. (2002). Complexity in conservation: Lessons from the global decline of amphibian populations. Ecology Letters, 5, 597–608.

[eva12940-bib-0017] Blaustein, A. R. , Urbina, J. , Snyder, P. W. , Reynolds, E. , Dang, T. , Hoverman, J. T. , … Hambalek, N. M. (2018). Effects of emerging infectious diseases on amphibians: A review of experimental studies. Diversity, 10, 81.

[eva12940-bib-0018] Bolliger, J. , Lander, T. , & Balkenhol, N. (2014). Landscape genetics since 2003: Status, challenges and future directions. Landscape Ecology, 29, 361–366.

[eva12940-bib-0019] Bosch, J. , Carrascal, L. M. , Duran, L. , Walker, S. , & Fisher, M. C. (2007). Climate change and outbreaks of amphibian chytridiomycosis in a montane area of Central Spain; is there a link? Proceedings of the Royal Society B‐Biological Sciences, 274, 253–260. 10.1098/rspb.2006.3713 PMC1685858 17148254

[eva12940-bib-0020] Bosch, J. , Fernández‐Beaskoetxea, S. , Garner, T. W. , & Carrascal, L. M. (2018). Long‐term monitoring of an amphibian community after a climate change‐and infectious disease‐driven species extirpation. Global Change Biology, 24, 2622–2632. 29446515 10.1111/gcb.14092

[eva12940-bib-0021] Bosch, J. , & Rincón, P. A. (2008). Chytridiomycosis‐mediated expansion of *Bufo bufo* in a montane area of Central Spain: An indirect effect of the disease. Diversity and Distributions, 14, 637–643.

[eva12940-bib-0022] Boyle, D. , Boyle, D. , Olsen, V. , Morgan, J. , & Hyatt, A. (2004). Rapid quantitative detection of chytridiomycosis (*Batrachochytrium dendrobatidis*) in amphibian samples using real‐time Taqman PCR assay. Diseases of Aquatic Organisms, 60, 141–148. 15460858 10.3354/dao060141

[eva12940-bib-0023] Brady, S. P. (2012). Road to evolution? Local adaptation to road adjacency in an amphibian (*Ambystoma maculatum*). Scientific Reports, 2, 235. 22355748 10.1038/srep00235 PMC3267261

[eva12940-bib-0024] Brown, R. M. , Linkem, C. W. , Siler, C. D. , Sukumaran, J. , Esselstyn, J. A. , Diesmos, A. C. , … McGuire, J. A. (2010). Phylogeography and historical demography of *Polypedates leucomystax* in the islands of Indonesia and the Philippines: Evidence for recent human‐mediated range expansion? Molecular Phylogenetics and Evolution, 57, 598–619. 20601009 10.1016/j.ympev.2010.06.015

[eva12940-bib-0025] Cadotte, M. W. , Carscadden, K. , & Mirotchnick, N. (2011). Beyond species: Functional diversity and the maintenance of ecological processes and services. Journal of Applied Ecology, 48, 1079–1087.

[eva12940-bib-0026] Campos, F. S. , Lourenço‐de‐Moraes, R. , Llorente, G. A. , & Solé, M. (2017). Cost‐effective conservation of amphibian ecology and evolution. Science Advances, 3, e1602929. 28691084 10.1126/sciadv.1602929 PMC5479652

[eva12940-bib-0027] Carroll, S. P. , Jørgensen, P. S. , Kinnison, M. T. , Bergstrom, C. T. , Denison, R. F. , Gluckman, P. , … Tabashnik, B. E. (2014). Applying evolutionary biology to address global challenges. Science, 346, 1245993. 25213376 10.1126/science.1245993 PMC4245030

[eva12940-bib-0028] Cayuela, H. , Valenzuela‐Sanchez, A. , Teulier, L. , Martínez‐Solano, Í. , Léna, J.‐P. , Merilä, J. , … Denoël, M. (2018). Determinants and consequences of dispersal in vertebrates with complex life cycles: A review of pond‐breeding amphibians. PeerJ, 6, e27394v1 Preprints. 10.7287/peerj.preprints.27394v1

[eva12940-bib-0029] Charlesworth, B. , & Charlesworth, D. (2010). Elements of evolutionary genetics. Greenwood Village, CO: Roberts.

[eva12940-bib-0030] Christie, M. R. , Marine, M. L. , French, R. A. , & Blouin, M. S. (2012). Genetic adaptation to captivity can occur in a single generation. Proceedings of the National Academy of Sciences, 109, 238–242. 10.1073/pnas.1111073109 PMC3252900 22184236

[eva12940-bib-0031] Christie, M. R. , & Searle, C. L. (2018). Evolutionary rescue in a host–pathogen system results in coexistence not clearance. Evolutionary Applications, 11, 681–693. 29875810 10.1111/eva.12568 PMC5979755

[eva12940-bib-0032] Cohen, J. M. , Civitello, D. J. , Venesky, M. D. , McMahon, T. A. , & Rohr, J. R. (2019). An interaction between climate change and infectious disease drove widespread amphibian declines. Global Change Biology, 25, 927–937. 30484936 10.1111/gcb.14489

[eva12940-bib-0033] Coles, R. S. , Reading, C. J. , & Jehle, R. (2019). Linking effective population size dynamics to phenotypic traits in the common toad (*Bufo bufo*). Conservation Genetics, 20, 987–995.

[eva12940-bib-0034] Collins, J. P. , & Crump, M. L. (2009). Extinction in our times: Global amphibian decline. New York, NY: Oxford University Press.

[eva12940-bib-0035] Cosentino, B. J. , Moore, J. D. , Karraker, N. E. , Ouellet, M. , & Gibbs, J. P. (2017). Evolutionary response to global change: Climate and land use interact to shape color polymorphism in a woodland salamander. Ecology and Evolution, 7, 5426–5434. 28770079 10.1002/ece3.3118 PMC5528218

[eva12940-bib-0036] Coster, S. S. , Babbitt, K. J. , & Kovach, A. I. (2015). High genetic connectivity in wood frogs (*Lithobates sylvaticus*) and spotted salamanders (*Ambystoma maculatum*) in a commercial forest. Herpetological Conservation and Biology, 10, 64–89.

[eva12940-bib-0037] Cushman, S. A. (2006). Effects of habitat loss and fragmentation on amphibians: A review and prospectus. Biological Conservation, 128, 231–240.

[eva12940-bib-0038] Davies, S. J. , Clusella‐Trullas, S. , Hui, C. , & McGeoch, M. A. (2013). Farm dams facilitate amphibian invasion: Extra‐limital range expansion of the painted reed frog in South Africa. Austral Ecology, 38, 851–863.

[eva12940-bib-0039] Davies, S. J. , Hill, M. P. , McGeoch, M. A. , & Clusella‐Trullas, S. (2019). Niche shift and resource supplementation facilitate an amphibian range expansion. Diversity and Distributions, 25, 154–165.

[eva12940-bib-0040] Díaz‐García, J. M. , Pineda, E. , López‐Barrera, F. , & Moreno, C. E. (2017). Amphibian species and functional diversity as indicators of restoration success in tropical montane forest. Biodiversity and Conservation, 26, 2569–2589.

[eva12940-bib-0041] Dudek, K. , Gaczorek, T. , Zieliński, P. , & Babik, W. (2019). Massive introgression of MHC genes in newt hybrid zones. Molecular Ecology, 28, 4798–4810. 31574568 10.1111/mec.15254

[eva12940-bib-0042] Duffus, A. L. , Waltzek, T. B. , Stöhr, A. C. , Allender, M. C. , Gotesman, M. , Whittington, R. J. , … Marschang, R. E. (2015). Distribution and host range of ranaviruses In Gray M. J., & Chinchar V. G., Ranaviruses (pp. 9–57). Cham, Switzerland: Springer.

[eva12940-bib-0043] Dufresnes, C. , Di Santo, L. , Leuenberger, J. , Schuerch, J. , Mazepa, G. , Grandjean, N. , … Dubey, S. (2017). Cryptic invasion of Italian pool frogs (*Pelophylax bergeri*) across Western Europe unraveled by multilocus phylogeography. Biological Invasions, 19, 1407–1420.

[eva12940-bib-0044] Dufresnes, C. , & Dubey, S. (2020). Invasion genomics supports an old hybrid swarm of pool frogs in Western Europe. Biological Invasions, 22, 205–210.

[eva12940-bib-0045] Dufresnes, C. , Pellet, J. , Bettinelli‐Riccardi, S. , Thiébaud, J. , Perrin, N. , & Fumagalli, L. (2016). Massive genetic introgression in threatened northern crested newts (*Triturus cristatus*) by an invasive congener (*T. carnifex*) in Western Switzerland. Conservation Genetics, 17, 839–846.

[eva12940-bib-0046] Ehrenreich, I. M. , & Pfennig, D. W. (2015). Genetic assimilation: A review of its potential proximate causes and evolutionary consequences. Annals of Botany, 117, 769–779. 26359425 10.1093/aob/mcv130 PMC4845796

[eva12940-bib-0047] Epps, C. W. , & Keyghobadi, N. (2015). Landscape genetics in a changing world: Disentangling historical and contemporary influences and inferring change. Molecular Ecology, 24, 6021–6040. 26547281 10.1111/mec.13454

[eva12940-bib-0048] Evans, A. E. , Forester, B. R. , Jockusch, E. L. , & Urban, M. C. (2018). Salamander morph frequencies do not evolve as predicted in response to 40 years of climate change. Ecography, 41, 1687–1697.

[eva12940-bib-0049] Ficetola, G. F. , Garner, T. W. , & De Bernardi, F. (2007). Genetic diversity, but not hatching success, is jointly affected by postglacial colonization and isolation in the threatened frog, *Rana latastei* . Molecular Ecology, 16, 1787–1797. 17444892 10.1111/j.1365-294X.2006.03198.x

[eva12940-bib-0050] Ficetola, G. F. , Garner, T. W. , Wang, J. , & De Bernardi, F. (2011). Rapid selection against inbreeding in a wild population of a rare frog. Evolutionary Applications, 4, 30–38. 25567951 10.1111/j.1752-4571.2010.00130.x PMC3352519

[eva12940-bib-0051] Fijarczyk, A. , Dudek, K. , Niedzicka, M. , & Babik, W. (2018). Balancing selection and introgression of newt immune‐response genes. Proceedings of the Royal Society of London B: Biological Sciences, 285, 20180819. 10.1098/rspb.2018.0819 PMC6111169 30111606

[eva12940-bib-0052] Fisher, M. C. , & Garner, T. W. (2007). The relationship between the emergence of *Batrachochytrium dendrobatidis*, the international trade in amphibians and introduced amphibian species. Fungal Biology Reviews, 21, 2–9.

[eva12940-bib-0053] Fites, J. S. , Ramsey, J. P. , Holden, W. M. , Collier, S. P. , Sutherland, D. M. , Reinert, L. K. , … Oswald‐Richter, K. (2013). The invasive chytrid fungus of amphibians paralyzes lymphocyte responses. Science, 342, 366–369. 24136969 10.1126/science.1243316 PMC3956111

[eva12940-bib-0054] Flynn, R. W. , Love, C. N. , Coleman, A. , & Lance, S. L. (2019). Variation in metal tolerance associated with population exposure history in Southern toads (*Anaxyrus terrestris*). Aquatic Toxicology, 207, 163–169. 30572176 10.1016/j.aquatox.2018.12.009

[eva12940-bib-0055] Frankham, R. (2005). Genetics and extinction. Biological Conservation, 126, 131–140.

[eva12940-bib-0056] Frankham, R. (2008). Genetic adaptation to captivity in species conservation programs. Molecular Ecology, 17, 325–333. 18173504 10.1111/j.1365-294X.2007.03399.x

[eva12940-bib-0057] Frankham, R. (2015). Genetic rescue of small inbred populations: Meta‐analysis reveals large and consistent benefits of gene flow. Molecular Ecology, 24, 2610–2618. 25740414 10.1111/mec.13139

[eva12940-bib-0058] Frankham, R. , Ballou, J. D. , & Briscoe, D. A. (2010). Introduction to conservation genetics. Cambridge: Cambridge University Press.

[eva12940-bib-0059] Frankham, R. , Ballou, J. D. , Eldridge, M. D. , Lacy, R. C. , Ralls, K. , Dudash, M. R. , & Fenster, C. B. (2011). Predicting the probability of outbreeding depression. Conservation Biology, 25, 465–475. 21486369 10.1111/j.1523-1739.2011.01662.x

[eva12940-bib-0060] Freidenburg, K. L. , & Skelly, D. K. (2004). Microgeographical variation in thermal preference by an amphibian. Ecology Letters, 7, 369–373.

[eva12940-bib-0061] Funk, W. C. , Zamudio, K. R. , & Crawford, A. J. (2018). Advancing understanding of amphibian evolution, ecology, behavior, and conservation with massively parallel sequencing In P. Hohenlohe, & O. P. Rajora (Eds.), Population genomics. (pp. 1‐44) Cham, Switzerland: Springer.

[eva12940-bib-0062] Germano, J. M. , & Bishop, P. J. (2009). Suitability of amphibians and reptiles for translocation. Conservation Biology, 23, 7–15. 19143783 10.1111/j.1523-1739.2008.01123.x

[eva12940-bib-0063] Gervasi, S. S. , Stephens, P. R. , Hua, J. , Searle, C. L. , Xie, G. Y. , Urbina, J. , … Hammond, J. I. (2017). Linking ecology and epidemiology to understand predictors of multi‐host responses to an emerging pathogen, the amphibian chytrid fungus. PLoS ONE, 12, e0167882. 28095428 10.1371/journal.pone.0167882 PMC5240985

[eva12940-bib-0064] Ghalambor, C. K. , McKay, J. K. , Carroll, S. P. , & Reznick, D. N. (2007). Adaptive versus non‐adaptive phenotypic plasticity and the potential for contemporary adaptation in new environments. Functional Ecology, 21, 394–407.

[eva12940-bib-0065] Goldberg, C. S. , Strickler, K. M. , & Fremier, A. K. (2018). Degradation and dispersion limit environmental DNA detection of rare amphibians in wetlands: Increasing efficacy of sampling designs. Science of the Total Environment, 633, 695–703. 29602110 10.1016/j.scitotenv.2018.02.295

[eva12940-bib-0066] González‐del‐Pliego, P. , Freckleton, R. P. , Edwards, D. P. , Koo, M. S. , Scheffers, B. R. , Pyron, R. A. , & Jetz, W. (2019). Phylogenetic and trait‐based prediction of extinction risk for data‐deficient amphibians. Current Biology, 29, 1557–1563. 31063716 10.1016/j.cub.2019.04.005

[eva12940-bib-0067] Gower, D. J. , & Wilkinson, M. (2005). Conservation biology of caecilian amphibians. Conservation Biology, 19, 45–55.

[eva12940-bib-0068] Grant, E. H. C. , Miller, D. A. , Schmidt, B. R. , Adams, M. J. , Amburgey, S. M. , Chambert, T. , … Hossack, B. R. (2016). Quantitative evidence for the effects of multiple drivers on continental‐scale amphibian declines. Scientific Reports, 6, 25625. 27212145 10.1038/srep25625 PMC4876446

[eva12940-bib-0069] Grant, E. H. C. , Muths, E. , Schmidt, B. R. , & Petrovan, S. O. (2019). Amphibian conservation in the Anthropocene. Biological Conservation, 236, 543–547.

[eva12940-bib-0070] Greenberg, D. , Palen, W. , Chan, K. , Jetz, W. , & Mooers, A. (2018). Evolutionarily distinct amphibians are disproportionately lost from human‐modified ecosystems. Ecology Letters, 21, 1530–1540. 30133091 10.1111/ele.13133

[eva12940-bib-0071] Greenspan, S. , Lambertini, C. , Carvalho, T. , James, T. , Toledo, L. , Haddad, C. , & Becker, C. (2018). Hybrids of amphibian chytrid show high virulence in native hosts. Scientific Reports, 8, 9600. 29941894 10.1038/s41598-018-27828-w PMC6018099

[eva12940-bib-0072] Gruber, J. , Brown, G. , Whiting, M. J. , & Shine, R. (2017). Is the behavioural divergence between range‐core and range‐edge populations of cane toads (*Rhinella marina*) due to evolutionary change or developmental plasticity? Royal Society Open Science, 4, 170789. 29134082 10.1098/rsos.170789 PMC5666265

[eva12940-bib-0073] Gutiérrez‐Rodríguez, J. , Gonçalves, J. , Civantos, E. , & Martínez‐Solano, I. (2017). Comparative landscape genetics of pond‐breeding amphibians in Mediterranean temporal wetlands: The positive role of structural heterogeneity in promoting gene flow. Molecular Ecology, 26, 5407–5420. 28752597 10.1111/mec.14272

[eva12940-bib-0074] Haddad, N. M. , Brudvig, L. A. , Clobert, J. , Davies, K. F. , Gonzalez, A. , Holt, R. D. , … Collins, C. D. (2015). Habitat fragmentation and its lasting impact on Earth’s ecosystems. Science Advances, 1, e1500052. 26601154 10.1126/sciadv.1500052 PMC4643828

[eva12940-bib-0075] Halfwerk, W. , Blaas, M. , Kramer, L. , Hijner, N. , Trillo, P. A. , Bernal, X. E. , … Ellers, J. (2019). Adaptive changes in sexual signalling in response to urbanization. Nature Ecology & Evolution, 3, 374. 30532046 10.1038/s41559-018-0751-8

[eva12940-bib-0076] Halverson, M. , Skelly, D. , & Caccone, A. (2006). Inbreeding linked to amphibian survival in the wild but not in the laboratory. Journal of Heredity, 97, 499–507. 16957048 10.1093/jhered/esl019

[eva12940-bib-0077] Hamilton, J. A. , & Miller, J. M. (2016). Adaptive introgression as a resource for management and genetic conservation in a changing climate. Conservation Biology, 30, 33–41. 26096581 10.1111/cobi.12574

[eva12940-bib-0078] Harding, G. , Griffiths, R. A. , & Pavajeau, L. (2016). Developments in amphibian captive breeding and reintroduction programs. Conservation Biology, 30, 340–349. 26306460 10.1111/cobi.12612

[eva12940-bib-0079] Heard, G. W. , Scroggie, M. P. , & Malone, B. S. (2012). Classical metapopulation theory as a useful paradigm for the conservation of an endangered amphibian. Biological Conservation, 148, 156–166.

[eva12940-bib-0080] Hedrick, P. W. (2013). Adaptive introgression in animals: Examples and comparison to new mutation and standing variation as sources of adaptive variation. Molecular Ecology, 22, 4606–4618. 23906376 10.1111/mec.12415

[eva12940-bib-0081] Heinicke, M. P. , Diaz, L. M. , & Hedges, S. B. (2011). Origin of invasive Florida frogs traced to Cuba. Biology Letters, 7, 407–410. 21270024 10.1098/rsbl.2010.1131 PMC3097879

[eva12940-bib-0082] Hendry, A. P. (2016). Eco‐evolutionary dynamics. Princeton: Princeton University Press.

[eva12940-bib-0083] Hettyey, A. , Ujszegi, J. , Herczeg, D. , Holly, D. , Vörös, J. , Schmidt, B. R. , & Bosch, J. (2019). Taking advantage of the thermal optimum mismatch between a pathogen and its endangered hosts: The potential of localized heating in reducing prevalence and intensity of *Batrachochytrium dendrobatidis* infection in natural populations. Frontiers in Ecology and Evolution, 7, 254.

[eva12940-bib-0084] Hill, G. E. (2019). Mitonuclear ecology. Oxford: Oxford University Press.

[eva12940-bib-0085] Hinkson, K. M. , & Richter, S. C. (2016). Temporal trends in genetic data and effective population size support efficacy of management practices in critically endangered dusky gopher frogs (*Lithobates sevosus*). Ecology and Evolution, 6, 2667–2678. 27066242 10.1002/ece3.2084 PMC4798149

[eva12940-bib-0086] Hoffmann, A. A. , & Sgro, C. M. (2011). Climate change and evolutionary adaptation. Nature, 470, 479–485. 21350480 10.1038/nature09670

[eva12940-bib-0087] Hoffmann, A. A. , Sgrò, C. M. , & Kristensen, T. N. (2017). Revisiting adaptive potential, population size, and conservation. Trends in Ecology & Evolution, 32, 506–517. 28476215 10.1016/j.tree.2017.03.012

[eva12940-bib-0088] Homola, J. J. , Loftin, C. S. , Cammen, K. M. , Helbing, C. C. , Birol, I. , Schultz, T. F. , & Kinnison, M. T. (2019). Replicated landscape genomics identifies evidence of local adaptation to urbanization in wood frogs. Journal of Heredity, 110, 707–719. 31278891 10.1093/jhered/esz041 PMC6785938

[eva12940-bib-0089] Hopkins, G. R. , French, S. S. , & Brodie, E. D. Jr (2013). Potential for local adaptation in response to an anthropogenic agent of selection: Effects of road deicing salts on amphibian embryonic survival and development. Evolutionary Applications, 6, 384–392. 23467723 10.1111/eva.12016 PMC3586626

[eva12940-bib-0090] Horner, A. A. , Hoffman, E. A. , Tye, M. R. , Hether, T. D. , & Savage, A. E. (2017). Cryptic chytridiomycosis linked to climate and genetic variation in amphibian populations of the southeastern United States. PLoS ONE, 12, e0175843. 28448517 10.1371/journal.pone.0175843 PMC5407605

[eva12940-bib-0091] Hoverman, J. T. , Hoye, B. J. , & Johnson, P. T. (2013). Does timing matter? How priority effects influence the outcome of parasite interactions within hosts. Oecologia, 173, 1471–1480. 23754306 10.1007/s00442-013-2692-x

[eva12940-bib-0092] Hua, J. , Jones, D. K. , Mattes, B. M. , Cothran, R. D. , Relyea, R. A. , & Hoverman, J. T. (2015). The contribution of phenotypic plasticity to the evolution of insecticide tolerance in amphibian populations. Evolutionary Applications, 8, 586–596. 26136824 10.1111/eva.12267 PMC4479514

[eva12940-bib-0093] Isaac, N. J. , Redding, D. W. , Meredith, H. M. , & Safi, K. (2012). Phylogenetically‐informed priorities for amphibian conservation. PLoS ONE, 7, e43912. 22952807 10.1371/journal.pone.0043912 PMC3431382

[eva12940-bib-0094] Isselin‐Nondedeu, F. , Trochet, A. , Joubin, T. , Picard, D. , Etienne, R. , Le Chevalier, H. , … Ribéron, A. (2017). Spatial genetic structure of *Lissotriton helveticus* L. following the restoration of a forest ponds network. Conservation Genetics, 18, 853–866.

[eva12940-bib-0095] James, T. Y. , Toledo, L. F. , Rödder, D. , da Silva Leite, D. , Belasen, A. M. , Betancourt‐Román, C. M. , … Longo, A. V. (2015). Disentangling host, pathogen, and environmental determinants of a recently emerged wildlife disease: Lessons from the first 15 years of amphibian chytridiomycosis research. Ecology and Evolution, 5, 4079–4097. 26445660 10.1002/ece3.1672 PMC4588650

[eva12940-bib-0096] Jiménez, R. R. , & Sommer, S. (2017). The amphibian microbiome: Natural range of variation, pathogenic dysbiosis, and role in conservation. Biodiversity and Conservation, 26, 763–786.

[eva12940-bib-0097] Johansson, M. , Primmer, C. R. , & Merilä, J. (2007). Does habitat fragmentation reduce fitness and adaptability? A case study of the common frog (*Rana temporaria*). Molecular Ecology, 16, 2693–2700. 17594440 10.1111/j.1365-294X.2007.03357.x

[eva12940-bib-0098] Johnson, P. T. , Kellermanns, E. , & Bowerman, J. (2011). Critical windows of disease risk: Amphibian pathology driven by developmental changes in host resistance and tolerance. Functional Ecology, 25, 726–734.

[eva12940-bib-0099] Joseph, M. B. , & Knapp, R. A. (2018). Disease and climate effects on individuals drive post‐reintroduction population dynamics of an endangered amphibian. Ecosphere, 9, e02499.

[eva12940-bib-0100] Karwacki, E. E. , Atkinson, M. S. , Ossiboff, R. J. , & Savage, A. E. (2018). Novel quantitative PCR assay specific for the emerging *Perkinsea* amphibian pathogen reveals seasonal infection dynamics. Diseases of Aquatic Organisms, 129, 85–98. 29972369 10.3354/dao03239

[eva12940-bib-0101] Kelleher, S. R. , Silla, A. J. , & Byrne, P. G. (2018). Animal personality and behavioral syndromes in amphibians: A review of the evidence, experimental approaches, and implications for conservation. Behavioral Ecology and Sociobiology, 72, 79.

[eva12940-bib-0102] Kelly, E. , & Phillips, B. L. (2016). Targeted gene flow for conservation. Conservation Biology, 30, 259–267. 26332195 10.1111/cobi.12623

[eva12940-bib-0103] Knapp, R. A. , Fellers, G. M. , Kleeman, P. M. , Miller, D. A. , Vredenburg, V. T. , Rosenblum, E. B. , & Briggs, C. J. (2016). Large‐scale recovery of an endangered amphibian despite ongoing exposure to multiple stressors. Proceedings of the National Academy of Sciences, 113, 11889–11894. 10.1073/pnas.1600983113 PMC5081604 27698128

[eva12940-bib-0104] Koprivnikar, J. , Gibson, C. H. , & Redfern, J. C. (2012). Infectious personalities: Behavioural syndromes and disease risk in larval amphibians. Proceedings of the Royal Society B: Biological Sciences, 279, 1544–1550. 10.1098/rspb.2011.2156 PMC3282355 22090390

[eva12940-bib-0105] Kosch, T. , Silva, C. , Brannelly, L. , Roberts, A. , Lau, Q. , Marantelli, G. , … Skerratt, L. (2019). Genetic potential for disease resistance in critically endangered amphibians decimated by chytridiomycosis. Animal Conservation, 22, 238–250.

[eva12940-bib-0106] Kovach, R. P. , Luikart, G. , Lowe, W. H. , Boyer, M. C. , & Muhlfeld, C. C. (2016). Risk and efficacy of human‐enabled interspecific hybridization for climate‐change adaptation: Response to Hamilton and Miller (2016). Conservation Biology, 30, 428–430. 26918487 10.1111/cobi.12678

[eva12940-bib-0107] Kraaijeveld‐Smit, F. J. , Griffiths, R. A. , Moore, R. D. , & Beebee, T. J. (2006). Captive breeding and the fitness of reintroduced species: A test of the responses to predators in a threatened amphibian. Journal of Applied Ecology, 43, 360–365.

[eva12940-bib-0108] Kraus, F. (2008). Alien reptiles and amphibians: A scientific compendium and analysis (Vol. 4). Dordrecht, NL: Springer Science & Business Media.

[eva12940-bib-0109] Kraus, F. (2015). Impacts from invasive reptiles and amphibians. Annual Review of Ecology, Evolution, and Systematics, 46, 75–97.

[eva12940-bib-0110] Kriger, K. M. , & Hero, J. M. (2007). The chytrid fungus *Batrachochytrium dendrobatidis* is non‐randomly distributed across amphibian breeding habitats. Diversity and Distributions, 13, 781–788.

[eva12940-bib-0111] Lemmon, E. M. , & Lemmon, A. R. (2010). Reinforcement in chorus frogs: Lifetime fitness estimates including intrinsic natural selection and sexual selection against hybrids. Evolution, 64, 1748–1761. 20100218 10.1111/j.1558-5646.2010.00955.x

[eva12940-bib-0112] Levis, N. A. , & Pfennig, D. W. (2019). How stabilizing selection and nongenetic inheritance combine to shape the evolution of phenotypic plasticity. Journal of Evolutionary Biology, 32, 706–716. 30968503 10.1111/jeb.13475

[eva12940-bib-0113] Lewis, C. H. , Richards‐Zawacki, C. L. , Ibáñez, R. , Luedtke, J. , Voyles, J. , Houser, P. , & Gratwicke, B. (2019). Conserving Panamanian harlequin frogs by integrating captive‐breeding and research programs. Biological Conservation, 236, 180–187.

[eva12940-bib-0114] Lind, M. , & Johansson, F. (2011). Testing the role of phenotypic plasticity for local adaptation: Growth and development in time‐constrained *Rana temporaria* populations. Journal of Evolutionary Biology, 24, 2696–2704. 21954876 10.1111/j.1420-9101.2011.02393.x

[eva12940-bib-0115] Lindström, T. , Brown, G. P. , Sisson, S. A. , Phillips, B. L. , & Shine, R. (2013). Rapid shifts in dispersal behavior on an expanding range edge. Proceedings of the National Academy of Sciences, 110, 13452–13456. 10.1073/pnas.1303157110 PMC3746873 23898175

[eva12940-bib-0116] Lips, K. R. (2016). Overview of chytrid emergence and impacts on amphibians. Philosophical Transactions of the Royal Society B: Biological Sciences, 371, 20150465. 10.1098/rstb.2015.0465 PMC5095542 28080989

[eva12940-bib-0117] Longo, A. V. , Fleischer, R. C. , & Lips, K. R. (2019). Double trouble: Co‐infections of chytrid fungi will severely impact widely distributed newts. Biological Invasions, 21, 2233–2245.

[eva12940-bib-0118] Loreau, M. , & De Mazancourt, C. (2013). Biodiversity and ecosystem stability: A synthesis of underlying mechanisms. Ecology Letters, 16, 106–115. 23346947 10.1111/ele.12073

[eva12940-bib-0119] Louppe, V. , Courant, J. , & Herrel, A. (2017). Differences in mobility at the range edge of an expanding invasive population of *Xenopus laevis* in the west of France. Journal of Experimental Biology, 220, 278–283. 28100805 10.1242/jeb.146589

[eva12940-bib-0120] Lourenço, A. , Álvarez, D. , Wang, I. J. , & Velo‐Antón, G. (2017). Trapped within the city: Integrating demography, time since isolation and population‐specific traits to assess the genetic effects of urbanization. Molecular Ecology, 26, 1498–1514. 28099779 10.1111/mec.14019

[eva12940-bib-0121] Lourenço, A. , Gonçalves, J. , Carvalho, F. , Wang, I. J. , & Velo‐Antón, G. (2019). Comparative landscape genetics reveals the evolution of viviparity reduces genetic connectivity in fire salamanders. Molecular Ecology, 28, 4573–4591. 31541595 10.1111/mec.15249

[eva12940-bib-0122] Lourenço‐de‐Moraes, R. , Campos, F. S. , Ferreira, R. B. , Solé, M. , Beard, K. H. , & Bastos, R. P. (2019). Back to the future: Conserving functional and phylogenetic diversity in amphibian‐climate refuges. Biodiversity and Conservation, 28, 1049–1073.

[eva12940-bib-0123] Luquet, E. , David, P. , Léna, J. P. , Joly, P. , Konecny, L. , Dufresnes, C. , … Plénet, S. (2011). Heterozygosity–fitness correlations among wild populations of European tree frog (*Hyla arborea*) detect fixation load. Molecular Ecology, 20, 1877–1887. 21410805 10.1111/j.1365-294X.2011.05061.x

[eva12940-bib-0124] Luquet, E. , Lena, J.‐P. , David, P. , Prunier, J. , Joly, P. , Lengagne, T. , … Plénet, S. (2013). Within‐and among‐population impact of genetic erosion on adult fitness‐related traits in the European tree frog *Hyla arborea* . Heredity, 110, 347‐354. 23250010 10.1038/hdy.2012.110 PMC3607180

[eva12940-bib-0125] Mantyka‐Pringle, C. S. , Visconti, P. , Di Marco, M. , Martin, T. G. , Rondinini, C. , & Rhodes, J. R. (2015). Climate change modifies risk of global biodiversity loss due to land‐cover change. Biological Conservation, 187, 103–111.

[eva12940-bib-0126] McCartney‐Melstad, E. , & Shaffer, H. B. (2015). Amphibian molecular ecology and how it has informed conservation. Molecular Ecology, 24, 5084–5109. 26437125 10.1111/mec.13391

[eva12940-bib-0127] McCartney‐Melstad, E. , Vu, J. K. , & Shaffer, H. B. (2018). Genomic data recover previously undetectable fragmentation effects in an endangered amphibian. Molecular Ecology, 27, 4430–4443. 30307076 10.1111/mec.14892

[eva12940-bib-0128] McKnight, D. T. , Schwarzkopf, L. , Alford, R. A. , Bower, D. S. , & Zenger, K. R. (2017). Effects of emerging infectious diseases on host population genetics: A review. Conservation Genetics, 18, 1235–1245.

[eva12940-bib-0129] McMahon, T. A. , Sears, B. F. , Venesky, M. D. , Bessler, S. M. , Brown, J. M. , Deutsch, K. , … Young, S. (2014). Amphibians acquire resistance to live and dead fungus overcoming fungal immunosuppression. Nature, 511, 224–228. 25008531 10.1038/nature13491 PMC4464781

[eva12940-bib-0130] Measey, G. , Vimercati, G. , De Villiers, F. , Mokhatla, M. , Davies, S. , Thorp, C. , … Kumschick, S. (2016). A global assessment of alien amphibian impacts in a formal framework. Diversity and Distributions, 22, 970–981.

[eva12940-bib-0131] Meilink, W. R. , Arntzen, J. W. , van Delft, J. J. , & Wielstra, B. (2015). Genetic pollution of a threatened native crested newt species through hybridization with an invasive congener in the Netherlands. Biological Conservation, 184, 145–153.

[eva12940-bib-0132] Mendelson, J. R. , Whitfield, S. M. , & Sredl, M. J. (2019). A recovery engine strategy for amphibian conservation in the context of disease. Biological Conservation, 236, 188–191.

[eva12940-bib-0133] Merilä, J. , & Hendry, A. P. (2014). Climate change, adaptation, and phenotypic plasticity: The problem and the evidence. Evolutionary Applications, 7, 1–14. 24454544 10.1111/eva.12137 PMC3894893

[eva12940-bib-0134] Mims, M. C. , Kirk, E. E. H. , Lytle, D. A. , & Olden, J. D. (2018). Traits‐based approaches support the conservation relevance of landscape genetics. Conservation Genetics, 19, 17–26.

[eva12940-bib-0135] Mittan, C. S. , & Zamudio, K. R. (2019). Rapid adaptation to cold in the invasive cane toad *Rhinella marina* . Conservation Physiology, 7, coy075. 30800317 10.1093/conphys/coy075 PMC6379050

[eva12940-bib-0136] Moran, E. V. , & Alexander, J. M. (2014). Evolutionary responses to global change: lessons from invasive species. Ecology Letters, 17, 637–649. 24612028 10.1111/ele.12262

[eva12940-bib-0137] Nori, J. , Lemes, P. , Urbina‐Cardona, N. , Baldo, D. , Lescano, J. , & Loyola, R. (2015). Amphibian conservation, land‐use changes and protected areas: A global overview. Biological Conservation, 191, 367–374.

[eva12940-bib-0138] Nowakowski, A. J. , Frishkoff, L. O. , Thompson, M. E. , Smith, T. M. , & Todd, B. D. (2018). Phylogenetic homogenization of amphibian assemblages in human‐altered habitats across the globe. Proceedings of the National Academy of Sciences, 115, E3454–E3462. 10.1073/pnas.1714891115 PMC5899437 29555733

[eva12940-bib-0139] Nunes, A. L. , Fill, J. M. , Davies, S. J. , Louw, M. , Rebelo, A. D. , Thorp, C. J. , … Measey, J. (2019). A global meta‐analysis of the ecological impacts of alien species on native amphibians. Proceedings of the Royal Society B, 286, 20182528. 30963838 10.1098/rspb.2018.2528 PMC6408899

[eva12940-bib-0140] O’Donnell, R. P. , Drost, C. A. , & Mock, K. E. (2017). Cryptic invasion of Northern Leopard Frogs (*Rana pipiens*) across phylogeographic boundaries and a dilemma for conservation of a declining amphibian. Biological Invasions, 19, 1039–1052.

[eva12940-bib-0141] O’Hanlon, S. J. , Rieux, A. , Farrer, R. A. , Rosa, G. M. , Waldman, B. , Bataille, A. , … Fisher, M. C. (2018). Recent Asian origin of chytrid fungi causing global amphibian declines. Science, 360, 621–627. 29748278 10.1126/science.aar1965 PMC6311102

[eva12940-bib-0142] Olori, J. C. , Netzband, R. , McKean, N. , Lowery, J. , Parsons, K. , & Windstam, S. T. (2018). Multi‐year dynamics of ranavirus, chytridiomycosis, and co‐infections in a temperate host assemblage of amphibians. Diseases of Aquatic Organisms, 130, 187–197. 30259871 10.3354/dao03260

[eva12940-bib-0143] Pabijan, M. , Wollenberg, K. , & Vences, M. (2012). Small body size increases the regional differentiation of populations of tropical mantellid frogs (Anura: Mantellidae). Journal of Evolutionary Biology, 25, 2310–2324. 22998688 10.1111/j.1420-9101.2012.02613.x

[eva12940-bib-0144] Pabijan, M. , Zielinski, P. , Dudek, K. , Stuglik, M. , & Babik, W. (2017). Isolation and gene flow in a speciation continuum in newts. Molecular Phylogenetics and Evolution, 116, 1–12. 28797693 10.1016/j.ympev.2017.08.003

[eva12940-bib-0145] Paetow, L. J. , McLaughlin, J. D. , Pauli, B. D. , & Marcogliese, D. J. (2013). Mortality of American bullfrog tadpoles *Lithobates catesbeianus* infected by *Gyrodactylus jennyae* and experimentally exposed to *Batrachochytrium dendrobatidis* . Journal of Aquatic Animal Health, 25, 15–26. 23290030 10.1080/08997659.2012.722170

[eva12940-bib-0146] Palomar, G. , Bosch, J. , & Cano, J. M. (2016). Heritability of *Batrachochytrium dendrobatidis* burden and its genetic correlation with development time in a population of common toad (*Bufo spinosus*). Evolution, 70, 2346–2356. 27480345 10.1111/evo.13029

[eva12940-bib-0147] Parris, M. J. (2004). Hybrid response to pathogen infection in interspecific crosses between two amphibian species (Anura: Ranidae). Evolutionary Ecology Research, 6, 457–471.

[eva12940-bib-0148] Peacock, M. M. , Beard, K. H. , O’Neill, E. M. , Kirchoff, V. S. , & Peters, M. B. (2009). Strong founder effects and low genetic diversity in introduced populations of Coqui frogs. Molecular Ecology, 18, 3603–3615. 19674300 10.1111/j.1365-294X.2009.04308.x

[eva12940-bib-0149] Pearman, P. B. , & Garner, T. W. J. (2005). Susceptibility of Italian agile frog populations to an emerging strain of Ranavirus parallels population genetic diversity. Ecology Letters, 8, 401–408.

[eva12940-bib-0150] Perkins, A. T. , Phillips, B. L. , Baskett, M. L. , & Hastings, A. (2013). Evolution of dispersal and life history interact to drive accelerating spread of an invasive species. Ecology Letters, 16, 1079–1087. 23809102 10.1111/ele.12136

[eva12940-bib-0151] Perl, R. B. , Geffen, E. , Malka, Y. , Barocas, A. , Renan, S. , Vences, M. , & Gafny, S. (2018). Population genetic analysis of the recently rediscovered Hula painted frog (*Latonia nigriventer*) reveals high genetic diversity and low inbreeding. Scientific Reports, 8, 5588. 29615810 10.1038/s41598-018-23587-w PMC5882862

[eva12940-bib-0152] Phillips, B. , Brown, G. , & Shine, R. (2010). Evolutionarily accelerated invasions: The rate of dispersal evolves upwards during the range advance of cane toads. Journal of Evolutionary Biology, 23, 2595–2601. 20939838 10.1111/j.1420-9101.2010.02118.x

[eva12940-bib-0153] Phillips, B. L. , Brown, G. P. , Webb, J. K. , & Shine, R. (2006). Invasion and the evolution of speed in toads. Nature, 439, 803. 16482148 10.1038/439803a

[eva12940-bib-0154] Phillipsen, I. C. , Funk, W. C. , Hoffman, E. A. , Monsen, K. J. , & Blouin, M. S. (2011). Comparative analyses of effective population size within and among species: Ranid frogs as a case study. Evolution, 65, 2927–2945. 21967433 10.1111/j.1558-5646.2011.01356.x

[eva12940-bib-0155] Picco, A. M. , & Collins, J. P. (2008). Amphibian commerce as a likely source of pathogen pollution. Conservation Biology, 22, 1582–1589. 18717688 10.1111/j.1523-1739.2008.01025.x

[eva12940-bib-0156] Pierce, A. A. , Gutierrez, R. , Rice, A. M. , & Pfennig, K. S. (2017). Genetic variation during range expansion: Effects of habitat novelty and hybridization. Proceedings of the Royal Society B: Biological Sciences, 284, 20170007. 10.1098/rspb.2017.0007 PMC5394663 28381622

[eva12940-bib-0157] Quintero, I. , & Wiens, J. J. (2013). Rates of projected climate change dramatically exceed past rates of climatic niche evolution among vertebrate species. Ecology Letters, 16, 1095–1103. 23800223 10.1111/ele.12144

[eva12940-bib-0158] Rae, J. , & Murray, D. (2019). Pathogen vs. predator: Ranavirus exposure dampens tadpole responses to perceived predation risk. Oecologia, 191, 325–334. 31535255 10.1007/s00442-019-04501-1

[eva12940-bib-0159] Raffel, T. , Rohr, J. , Kiesecker, J. , & Hudson, P. J. (2006). Negative effects of changing temperature on amphibian immunity under field conditions. Functional Ecology, 20, 819–828.

[eva12940-bib-0160] Ralls, K. , Ballou, J. D. , Dudash, M. R. , Eldridge, M. D. , Fenster, C. B. , Lacy, R. C. , … Frankham, R. (2018). Call for a paradigm shift in the genetic management of fragmented populations. Conservation Letters, 11, e12412.

[eva12940-bib-0161] Räsanen, K. , Laurila, A. , & Merilä, J. (2003). Geographic variation in acid stress tolerance of the moor frog, *Rana arvalis*. I. Local adaptation. Evolution, 57, 352–362. 12683531

[eva12940-bib-0162] Razgour, O. , Forester, B. , Taggart, J. B. , Bekaert, M. , Juste, J. , Ibáñez, C. , … Manel, S. (2019). Considering adaptive genetic variation in climate change vulnerability assessment reduces species range loss projections. Proceedings of the National Academy of Sciences, 116, 10418–10423. 10.1073/pnas.1820663116 PMC6535011 31061126

[eva12940-bib-0163] Redding, D. W. , & Mooers, A. Ø. (2006). Incorporating evolutionary measures into conservation prioritization. Conservation Biology, 20, 1670–1678. 17181802 10.1111/j.1523-1739.2006.00555.x

[eva12940-bib-0164] Restif, O. , & Koella, J. C. (2004). Concurrent evolution of resistance and tolerance to pathogens. The American Naturalist, 164, E90–E102. 10.1086/423713 15459887

[eva12940-bib-0165] Ribeiro, L. P. , Carvalho, T. , Becker, C. G. , Jenkinson, T. S. , da Silva Leite, D. , James, T. Y. , … Toledo, L. F. (2019). Bullfrog farms release virulent zoospores of the frog‐killing fungus into the natural environment. Scientific Reports, 9, 13422. 31530868 10.1038/s41598-019-49674-0 PMC6748994

[eva12940-bib-0166] Richardson, J. L. , Brady, S. P. , Wang, I. J. , & Spear, S. F. (2016). Navigating the pitfalls and promise of landscape genetics. Molecular Ecology, 25, 849–863. 26756865 10.1111/mec.13527

[eva12940-bib-0167] Richards‐Zawacki, C. L. (2009). Thermoregulatory behaviour affects prevalence of chytrid fungal infection in a wild population of Panamanian golden frogs. Proceedings of the Royal Society B: Biological Sciences, 277, 519–528. 10.1098/rspb.2009.1656 PMC2842693 19864287

[eva12940-bib-0168] Riemann, J. C. , Ndriantsoa, S. H. , Rödel, M.‐O. , & Glos, J. (2017). Functional diversity in a fragmented landscape—Habitat alterations affect functional trait composition of frog assemblages in Madagascar. Global Ecology and Conservation, 10, 173–183.

[eva12940-bib-0169] Rivera‐Ortíz, F. , Aguilar, R. , Arizmendi, M. , Quesada, M. , & Oyama, K. (2015). Habitat fragmentation and genetic variability of tetrapod populations. Animal Conservation, 18, 249–258.

[eva12940-bib-0170] Robertson, J. M. , Murphy, M. A. , Pearl, C. A. , Adams, M. J. , Páez‐Vacas, M. I. , Haig, S. M. , … Funk, W. C. (2018). Regional variation in drivers of connectivity for two frog species (*Rana pretiosa* and *R. luteiventris*) from the US Pacific Northwest. Molecular Ecology, 27, 3242–3256. 10.1111/mec.14798 30010212

[eva12940-bib-0171] Rollins, L. A. , Richardson, M. F. , & Shine, R. (2015). A genetic perspective on rapid evolution in cane toads (*Rhinella marina*). Molecular Ecology, 24, 2264–2276. 25894012 10.1111/mec.13184

[eva12940-bib-0172] Rosa, G. M. , Bosch, J. , Martel, A. , Pasmans, F. , Rebelo, R. , Griffiths, R. A. , & Garner, T. W. (2019). Sex‐biased disease dynamics increase extinction risk by impairing population recovery. Animal Conservation, 22, 579–588.

[eva12940-bib-0173] Rowe, G. , & Beebee, T. J. (2003). Population on the verge of a mutational meltdown? Fitness costs of genetic load for an amphibian in the wild. Evolution, 57, 177–181. 12643580 10.1111/j.0014-3820.2003.tb00228.x

[eva12940-bib-0174] Rowley, J. J. , & Alford, R. A. (2013). Hot bodies protect amphibians against chytrid infection in nature. Scientific Reports, 3, 1515. 23519020 10.1038/srep01515 PMC3604863

[eva12940-bib-0175] Roy, B. , & Kirchner, J. (2000). Evolutionary dynamics of pathogen resistance and tolerance. Evolution, 54, 51–63. 10937183 10.1111/j.0014-3820.2000.tb00007.x

[eva12940-bib-0176] Ryan, M. E. , Johnson, J. R. , & Fitzpatrick, B. M. (2009). Invasive hybrid tiger salamander genotypes impact native amphibians. Proceedings of the National Academy of Sciences, 106, 11166–11171. 10.1073/pnas.0902252106 PMC2703667 19564601

[eva12940-bib-0177] Sauer, E. L. , Fuller, R. C. , Richards‐Zawacki, C. L. , Sonn, J. , Sperry, J. H. , & Rohr, J. R. (2018). Variation in individual temperature preferences, not behavioural fever, affects susceptibility to chytridiomycosis in amphibians. Proceedings of the Royal Society B: Biological Sciences, 285, 20181111. 10.1098/rspb.2018.1111 PMC6125923 30135162

[eva12940-bib-0178] Sauer, E. L. , Trejo, N. , Hoverman, J. T. , & Rohr, J. R. (2019). Behavioural fever reduces ranaviral infection in toads. Functional Ecology, 33, 2172–2179. 10.1111/1365-2435.13427 PMC7546308 33041425

[eva12940-bib-0179] Savage, A. E. , Becker, C. G. , & Zamudio, K. R. (2015). Linking genetic and environmental factors in amphibian disease risk. Evolutionary Applications, 8, 560–572. 26136822 10.1111/eva.12264 PMC4479512

[eva12940-bib-0180] Savage, A. E. , & Zamudio, K. R. (2016). Adaptive tolerance to a pathogenic fungus drives major histocompatibility complex evolution in natural amphibian populations. Proceedings of the Royal Society B: Biological Sciences, 283, 20153115. 10.1098/rspb.2015.3115 PMC4822461 27009220

[eva12940-bib-0181] Savage, W. K. , Fremier, A. K. , & Bradley Shaffer, H. (2010). Landscape genetics of alpine Sierra Nevada salamanders reveal extreme population subdivision in space and time. Molecular Ecology, 19, 3301–3314. 20701683 10.1111/j.1365-294X.2010.04718.x

[eva12940-bib-0182] Scheele, B. C. , Pasmans, F. , Skerratt, L. F. , Berger, L. , Martel, A. , Beukema, W. , … Catenazzi, A. (2019). Amphibian fungal panzootic causes catastrophic and ongoing loss of biodiversity. Science, 363, 1459–1463. 30923224 10.1126/science.aav0379

[eva12940-bib-0183] Schmeller, D. S. , & Merilä, J. (2007). Demographic and genetic estimates of effective population and breeding size in the amphibian *Rana temporaria* . Conservation Biology, 21, 142–151. 17298520 10.1111/j.1523-1739.2006.00554.x

[eva12940-bib-0184] Seimon, T. A. , Seimon, A. , Daszak, P. , Halloy, S. R. , Schloegel, L. M. , Aguilar, C. A. , … E Simmons, J. (2007). Upward range extension of Andean anurans and chytridiomycosis to extreme elevations in response to tropical deglaciation. Global Change Biology, 13, 288–299.

[eva12940-bib-0185] Selechnik, D. , Richardson, M. F. , Shine, R. , DeVore, J. , Ducatez, S. , & Rollins, L. A. (2019). Bottleneck revisited: Increased adaptive variation despite reduced overall genetic diversity in a rapidly adapting invader. bioRxiv 557868 10.1101/557868 PMC6901984 31850072

[eva12940-bib-0186] Shaffer, H. B. , Gidiş, M. , McCartney‐Melstad, E. , Neal, K. M. , Oyamaguchi, H. M. , Tellez, M. , & Toffelmier, E. M. (2015). Conservation genetics and genomics of amphibians and reptiles. Annual Review of Animal Biosciences, 3, 113–138. 25580719 10.1146/annurev-animal-022114-110920

[eva12940-bib-0187] Shine, R. (2012). Invasive species as drivers of evolutionary change: Cane toads in tropical Australia. Evolutionary Applications, 5, 107–116. 25568034 10.1111/j.1752-4571.2011.00201.x PMC3353345

[eva12940-bib-0188] Shine, R. , Brown, G. P. , & Phillips, B. L. (2011). An evolutionary process that assembles phenotypes through space rather than through time. Proceedings of the National Academy of Sciences, 108, 5708–5711. 10.1073/pnas.1018989108 PMC3078378 21436040

[eva12940-bib-0189] Silla, A. J. , & Byrne, P. G. (2019). The role of reproductive technologies in amphibian conservation breeding programs. Annual Review of Animal Biosciences, 7, 499–519. 30359086 10.1146/annurev-animal-020518-115056

[eva12940-bib-0190] Skelly, D. , & Freidenburg, L. (2000). Effects of beaver on the thermal biology of an amphibian. Ecology Letters, 3, 483–486.

[eva12940-bib-0191] Skelly, D. K. , Joseph, L. N. , Possingham, H. P. , Freidenburg, L. K. , Farrugia, T. J. , Kinnison, M. T. , & Hendry, A. P. (2007). Evolutionary responses to climate change. Conservation Biology, 21, 1353–1355. 17883501 10.1111/j.1523-1739.2007.00764.x

[eva12940-bib-0192] Smith, M. A. , & Green, D. M. (2005). Dispersal and the metapopulation paradigm in amphibian ecology and conservation: Are all amphibian populations metapopulations? Ecography, 28, 110–128.

[eva12940-bib-0193] Spurgin, L. G. , & Richardson, D. S. (2010). How pathogens drive genetic diversity: MHC, mechanisms and misunderstandings. Proceedings of the Royal Society B: Biological Sciences, 277, 979–988. 10.1098/rspb.2009.2084 PMC2842774 20071384

[eva12940-bib-0194] Sterrett, S. C. , Katz, R. A. , Brand, A. B. , Fields, W. R. , Dietrich, A. E. , Hocking, D. J. , … Grant, E. H. C. (2019). Proactive management of amphibians: Challenges and opportunities. Biological Conservation, 236, 404–410.

[eva12940-bib-0195] Stockwell, C. A. , Hendry, A. P. , & Kinnison, M. T. (2003). Contemporary evolution meets conservation biology. Trends in Ecology & Evolution, 18, 94–101.

[eva12940-bib-0196] Stutz, W. E. , Blaustein, A. R. , Briggs, C. J. , Hoverman, J. T. , Rohr, J. R. , & Johnson, P. T. (2018). Using multi‐response models to investigate pathogen coinfections across scales: Insights from emerging diseases of amphibians. Methods in Ecology and Evolution, 9, 1109–1120. 29861885 10.1111/2041-210X.12938 PMC5978769

[eva12940-bib-0197] Teacher, A. G. , Garner, T. W. , & Nichols, R. A. (2009a). Evidence for directional selection at a novel major histocompatibility class I marker in wild common frogs (*Rana temporaria*) exposed to a viral pathogen (Ranavirus). PLoS ONE, 4, e4616. 19240796 10.1371/journal.pone.0004616 PMC2643007

[eva12940-bib-0198] Teacher, A. G. , Garner, T. W. , & Nichols, R. A. (2009b). Population genetic patterns suggest a behavioural change in wild common frogs (*Rana temporaria*) following disease outbreaks (Ranavirus). Molecular Ecology, 18, 3163–3172. 19566676 10.1111/j.1365-294X.2009.04263.x

[eva12940-bib-0199] Tennessen, J. B. , Parks, S. E. , Swierk, L. , Reinert, L. K. , Holden, W. M. , Rollins‐Smith, L. A. , … Langkilde, T. (2018). Frogs adapt to physiologically costly anthropogenic noise. Proceedings of the Royal Society B, 285, 20182194. 30464067 10.1098/rspb.2018.2194 PMC6253376

[eva12940-bib-0200] Tingley, R. , Ward‐Fear, G. , Schwarzkopf, L. , Greenlees, M. J. , Phillips, B. L. , Brown, G. , … Sheppard, A. (2017). New weapons in the Toad Toolkit: A review of methods to control and mitigate the biodiversity impacts of invasive cane toads (*Rhinella marina*). The Quarterly Review of Biology, 92, 123–149. 29562120 10.1086/692167

[eva12940-bib-0201] Todesco, M. , Pascual, M. A. , Owens, G. L. , Ostevik, K. L. , Moyers, B. T. , Hübner, S. , … Bock, D. G. (2016). Hybridization and extinction. Evolutionary Applications, 9, 892–908. 27468307 10.1111/eva.12367 PMC4947151

[eva12940-bib-0202] Tolley, K. A. , Davies, S. J. , & Chown, S. L. (2008). Deconstructing a controversial local range expansion: Conservation biogeography of the painted reed frog (*Hyperolius marmoratus*) in South Africa. Diversity and Distributions, 14, 400–411.

[eva12940-bib-0203] Urban, M. C. , Phillips, B. L. , Skelly, D. K. , & Shine, R. (2008). A toad more traveled: The heterogeneous invasion dynamics of cane toads in Australia. The American Naturalist, 171, E134–E148. 10.1086/527494 18271722

[eva12940-bib-0204] Urban, M. C. , Richardson, J. L. , & Freidenfelds, N. A. (2014). Plasticity and genetic adaptation mediate amphibian and reptile responses to climate change. Evolutionary Applications, 7, 88–103. 24454550 10.1111/eva.12114 PMC3894900

[eva12940-bib-0205] Urban, M. C. , Richardson, J. L. , Freidenfelds, N. A. , Drake, D. L. , Fischer, J. F. , & Saunders, P. P. (2017). Microgeographic adaptation of Wood Frog tadpoles to an apex predator. Copeia, 105, 451–461.

[eva12940-bib-0206] Valbuena‐Ureña, E. , Soler‐Membrives, A. , Steinfartz, S. , Orozco‐terWengel, P. , & Carranza, S. (2017). No signs of inbreeding despite long‐term isolation and habitat fragmentation in the critically endangered Montseny brook newt (*Calotriton arnoldi*). Heredity, 118, 424–435. 28074844 10.1038/hdy.2016.123 PMC5520529

[eva12940-bib-0207] Vences, M. , & Wake, D. B. (2007). Speciation, species boundaries and phylogeography of amphibians In Heatwole H. & Tyler M. (Eds.), Amphibian biology, Vol. 6, systematics (pp. 2613–2669). Chipping Norton, NSW: Surrey Beatty & Sons.

[eva12940-bib-0208] Venesky, M. D. , Raffel, T. R. , McMahon, T. A. , & Rohr, J. R. (2014). Confronting inconsistencies in the amphibian‐chytridiomycosis system: Implications for disease management. Biological Reviews, 89, 477–483. 24118903 10.1111/brv.12064

[eva12940-bib-0209] Vieites, D. R. , Wollenberg, K. C. , Andreone, F. , Köhler, J. , Glaw, F. , & Vences, M. (2009). Vast underestimation of Madagascar's biodiversity evidenced by an integrative amphibian inventory. Proceedings of the National Academy of Sciences, 106, 8267–8272. 10.1073/pnas.0810821106 PMC2688882 19416818

[eva12940-bib-0210] Vimercati, G. , Davies, S. J. , & Measey, J. (2018). Rapid adaptive response to a mediterranean environment reduces phenotypic mismatch in a recent amphibian invader. Journal of Experimental Biology, 221, jeb174797. 29615531 10.1242/jeb.174797

[eva12940-bib-0211] Vimercati, G. , Davies, S. J. , & Measey, J. (2019). Invasive toads adopt marked capital breeding when introduced to a cooler, more seasonal environment. Biological Journal of the Linnean Society, 128, 657–671.

[eva12940-bib-0212] Vogel, L. S. , & Johnson, S. G. (2008). Estimation of hybridization and introgression frequency in toads (genus: *Bufo*) using DNA sequence variation at mitochondrial and nuclear loci. Journal of Herpetology, 42, 61–76.

[eva12940-bib-0213] Voyles, J. , Woodhams, D. C. , Saenz, V. , Byrne, A. Q. , Perez, R. , Rios‐Sotelo, G. , … McLetchie, S. (2018). Shifts in disease dynamics in a tropical amphibian assemblage are not due to pathogen attenuation. Science, 359, 1517–1519. 29599242 10.1126/science.aao4806

[eva12940-bib-0214] Wang, J. , Santiago, E. , & Caballero, A. (2016). Prediction and estimation of effective population size. Heredity, 117, 193–206. 27353047 10.1038/hdy.2016.43 PMC5026755

[eva12940-bib-0215] Warne, R. W. , LaBumbard, B. , LaGrange, S. , Vredenburg, V. T. , & Catenazzi, A. (2016). Co‐infection by chytrid fungus and ranaviruses in wild and harvested frogs in the tropical Andes. PLoS ONE, 11, e0145864. 26726999 10.1371/journal.pone.0145864 PMC4701007

[eva12940-bib-0216] Wayne, R. K. , & Shaffer, H. B. (2016). Hybridization and endangered species protection in the molecular era. Molecular Ecology, 25, 2680–2689. 27064931 10.1111/mec.13642

[eva12940-bib-0217] Webb, C. O. , Ackerly, D. D. , McPeek, M. A. , & Donoghue, M. J. (2002). Phylogenies and community ecology. Annual Review of Ecology and Systematics, 33, 475–505.

[eva12940-bib-0218] Wielstra, B. , Babik, W. , & Arntzen, J. W. (2015). The crested newt *Triturus cristatus* recolonized temperate Eurasia from an extra‐Mediterranean glacial refugium. Biological Journal of the Linnean Society, 114, 574–587.

[eva12940-bib-0219] Wilber, M. Q. , Knapp, R. A. , Toothman, M. , & Briggs, C. J. (2017). Resistance, tolerance and environmental transmission dynamics determine host extinction risk in a load‐dependent amphibian disease. Ecology Letters, 20, 1169–1181. 28745026 10.1111/ele.12814

[eva12940-bib-0220] Willoughby, J. R. , & Christie, M. R. (2019). Long‐term demographic and genetic effects of releasing captive‐born individuals into the wild. Conservation Biology, 33, 377–388. 30168872 10.1111/cobi.13217

[eva12940-bib-0221] Wineland, S. M. , Arrick, R. F. , Welch, S. M. , Pauley, T. K. , Mosher, J. J. , Apodaca, J. J. , … Waldron, J. L. (2019). Environmental DNA improves Eastern Hellbender (*Cryptobranchus alleganiensis alleganiensis*) detection over conventional sampling methods. Environmental DNA, 1, 86–96.

[eva12940-bib-0222] Woodhams, D. C. , Alford, R. A. , Briggs, C. J. , Johnson, M. , & Rollins‐Smith, L. A. (2008). Life‐history trade‐offs influence disease in changing climates: Strategies of an amphibian pathogen. Ecology, 89, 1627–1639. 18589527 10.1890/06-1842.1

[eva12940-bib-0223] Woodhams, D. C. , Bosch, J. , Briggs, C. J. , Cashins, S. , Davis, L. R. , Lauer, A. , … Sheafor, B. (2011). Mitigating amphibian disease: Strategies to maintain wild populations and control chytridiomycosis. Frontiers in Zoology, 8, 8. 21496358 10.1186/1742-9994-8-8 PMC3098159

[eva12940-bib-0224] Wu, N. C. , Cramp, R. L. , & Franklin, C. E. (2018). Body size influences energetic and osmoregulatory costs in frogs infected with *Batrachochytrium dendrobatidis* . Scientific Reports, 8, 3739. 29487313 10.1038/s41598-018-22002-8 PMC5829222

[eva12940-bib-0225] Wuerthner, V. P. , Hua, J. , & Hoverman, J. T. (2017). The benefits of coinfection: Trematodes alter disease outcomes associated with virus infection. Journal of Animal Ecology, 86, 921–931. 28317105 10.1111/1365-2656.12665

[eva12940-bib-0226] Zeisset, I. , & Beebee, T. J. C. (2008). Amphibian phylogeography: A model for understanding historical aspects of species distributions. Heredity, 101, 109–119. 18493262 10.1038/hdy.2008.30

[eva12940-bib-0227] Zeisset, I. , & Beebee, T. (2013). Donor population size rather than local adaptation can be a key determinant of amphibian translocation success. Animal Conservation, 16, 359–366.

[eva12940-bib-0228] Zeisset, I. , & Hoogesteger, T. (2018). A reassessment of the biogeographic range of northern clade pool frogs (*Pelophylax lessonae*). Herpetological Journal, 28, 63–72.

